# Microbial Community Structure of Leaf-Cutter Ant Fungus Gardens and Refuse Dumps

**DOI:** 10.1371/journal.pone.0009922

**Published:** 2010-03-29

**Authors:** Jarrod J. Scott, Kevin J. Budsberg, Garret Suen, Devin L. Wixon, Teri C. Balser, Cameron R. Currie

**Affiliations:** 1 United States Department of Energy (DOE) Great Lakes Bioenergy Research Center, University of Wisconsin–Madison, Madison, Wisconsin, United States of America; 2 Department of Bacteriology, University of Wisconsin–Madison, Madison, Wisconsin, United States of America; 3 Smithsonian Tropical Research Institute (STRI), Balboa, Ancon, Republic of Panamá; 4 Department of Soil Science, University of Wisconsin–Madison, Madison, Wisconsin, United States of America; 5 Department of Botany, University of Wisconsin–Madison, Madison, Wisconsin, United States of America; University of Wisconsin-Milwaukee, United States of America

## Abstract

**Background:**

Leaf-cutter ants use fresh plant material to grow a mutualistic fungus that serves as the ants' primary food source. Within fungus gardens, various plant compounds are metabolized and transformed into nutrients suitable for ant consumption. This symbiotic association produces a large amount of refuse consisting primarily of partly degraded plant material. A leaf-cutter ant colony is thus divided into two spatially and chemically distinct environments that together represent a plant biomass degradation gradient. Little is known about the microbial community structure in gardens and dumps or variation between lab and field colonies.

**Methodology/Principal Findings:**

Using microbial membrane lipid analysis and a variety of community metrics, we assessed and compared the microbiota of fungus gardens and refuse dumps from both laboratory-maintained and field-collected colonies. We found that gardens contained a diverse and consistent community of microbes, dominated by Gram-negative bacteria, particularly γ-Proteobacteria and Bacteroidetes. These findings were consistent across lab and field gardens, as well as host ant taxa. In contrast, dumps were enriched for Gram-positive and anaerobic bacteria. Broad-scale clustering analyses revealed that community relatedness between samples reflected system component (gardens/dumps) rather than colony source (lab/field). At finer scales samples clustered according to colony source.

**Conclusions/Significance:**

Here we report the first comparative analysis of the microbiota from leaf-cutter ant colonies. Our work reveals the presence of two distinct communities: one in the fungus garden and the other in the refuse dump. Though we find some effect of colony source on community structure, our data indicate the presence of consistently associated microbes within gardens and dumps. Substrate composition and system component appear to be the most important factor in structuring the microbial communities. These results thus suggest that resident communities are shaped by the plant degradation gradient created by ant behavior, specifically their fungiculture and waste management.

## Introduction

**Figure 1:**
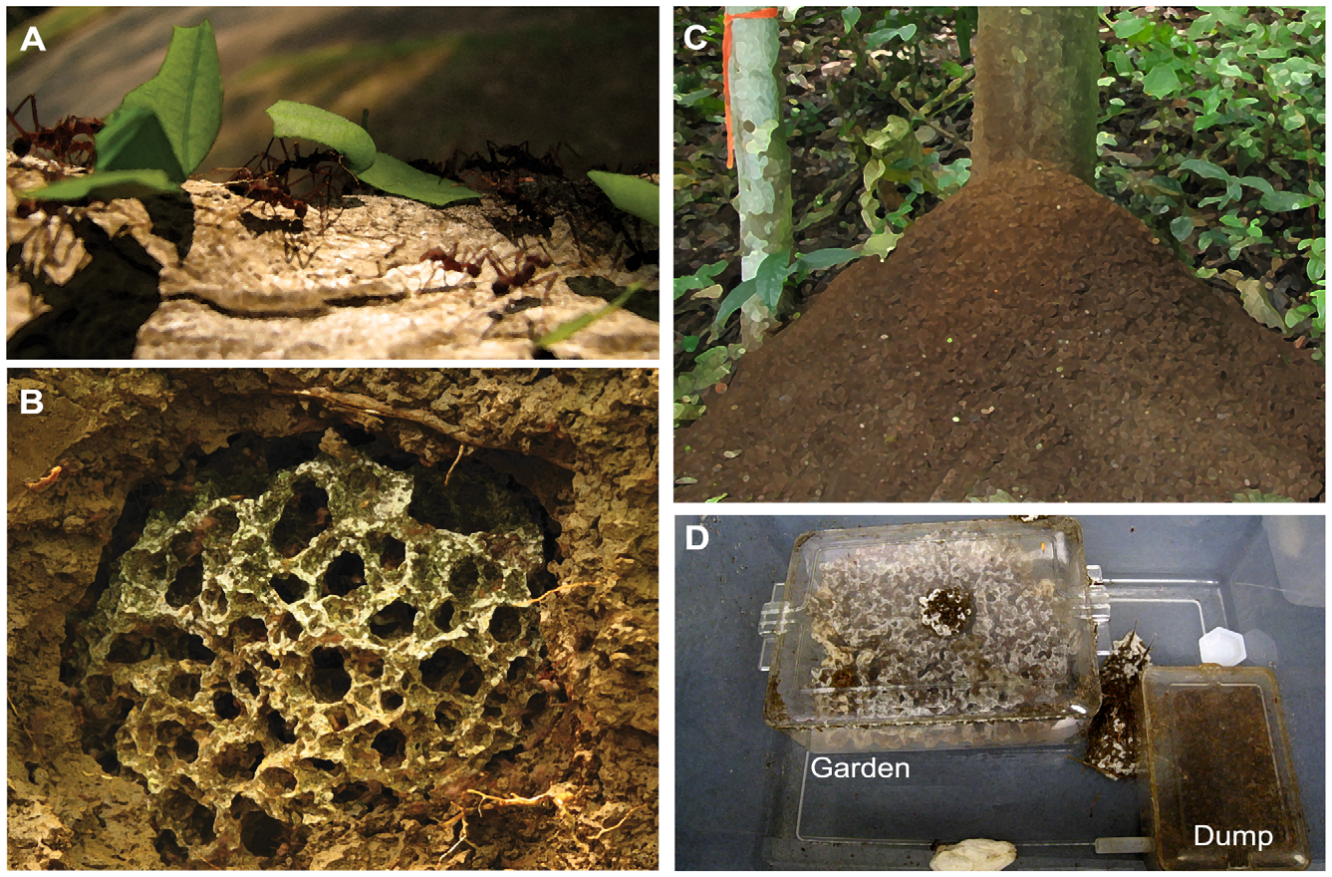
Representation of the leaf-cutter ant system. (**A**) Fresh plant material is harvested by foraging workers and brought back to the nest. (**B**) Plant material is processed and incorporated into the top of the garden, where it serves as the primary growth substrate for the mutualistic fungus. Substrate processing occurs over the course of several weeks and enzymatic analyses suggest that the fungus garden contains a decompositional gradient where more easily utilized material is extracted at the top of the garden and more recalcitrant material remains and appears to be partailly degraded at the garden bottom [12], [13]. (**C**) Older substrate and spent fungal material are removed from the bottom of the garden and transported to a refuse dump. Older workers manipulate material on the refuse dump presuambly to facilitate dgradation of the material [15]. (**D**) Typical lab colony set-up showing the relative orientation of the fungus garden and refuse dump. Gardens and dumps are housed in individual chambers within a larger box.

Leaf-cutter ants (*Atta* and *Acromyrmex*: Tribe Attini) are the most phylogenetically derived members of a monophyletic group of Neotropical ants that cultivate specialized fungal symbionts that serve as the colony's primary food source. Unlike other fungus-growing ants, leaf-cutters are unique in that they exclusively use fresh plant material to cultivate their fungal mutualist [1]–[4]. The ants protect their fungal cultivar from pathogens and parasites [3], [5]–[7], provide the fungus with a constant source of nutrients [3], [4], and aid in its growth and dispersal [3]. Fungiculture in attine ants originated once approximately 50 million years ago while the transition to fungal cultivation using fresh plant material appears to have arisen about 40 million years later [8]. Leaf-cutters are one of the most dominant herbivores in their native habitats [3], [4], with mature colonies of *Atta* potentially containing millions of workers living in an elaborate subterranean system of hundreds of interconnected fungal chambers [4]. Colonies can harvest a large amount of plant material, for example a study of *Atta colombica* colonies in Panama found that they collected an average of ∼250 Kg (dry weight) of plant material (including leaves, fruit, nuts, and flowers) per colony during a one-year observation period [9], [10]. Though a single colony will utilize up to 50% of the available local plant species, the bulk of plant material is harvested from relatively few plant species [9], [11] (Figure 1A). Foraging workers harvest fresh plant material that is brought back to the nest and incorporated into the top of the garden. To promote the initial degradation of plant biomass this material is masticated, mixed with ant fecal droplets, and inoculated with fungal mycelia [3] (Figure 1B).

The process of substrate addition and fungal planting is continuous: over time, the top layer of the garden is replaced by new plant material and gradually moves to the bottom of the garden matrix. Studies suggest that the more easily degraded plant compounds, such as starch and pectin, are extracted in the top of the garden [12]. In addition, degradation of hemicelluloses [12] and cellulose also occur throughout the fungus garden, while lignin appears to pass through undegraded [13]. Partially degraded plant material is removed from the bottom of the garden and deposited in refuse repositories (dumps) by workers. Most leaf-cutter ant species excavate specialized subterranean refuse chambers [3], [14], while a few deposit their waste in specialized and localized aboveground piles [3], [15] (Figure 1C). Mature colonies produce large amounts of refuse material [16], and considerable colony resources are devoted to waste management [15]. In addition, refuse dumps also provide important nutrient reservoirs for surrounding plant and animal communities [17]–[19].

Thus, the leaf-cutter ant garden and dump system represents a plant-based biomass degradation gradient, where the flow of plant-derived nutrients is mediated by fungiculture and waste management. These behaviors result in the concentration and separation of nutrients into two spatially distinct elements: the fungus garden and the refuse dump. Fungus gardens receive continual inputs of fresh plant material, while garden waste serves as the principal inocula for the dumps. Given the different degree of degradation between gardens and dumps, these environments differ markedly in the relative amounts of many plant polysaccharides and available nutrients. Such nutrient gradients may create spatial structuring for unique and specialized microbial communities. Although many studies have demonstrated that fungus gardens can harbor a variety of microorganisms [20]–[26], it remains unknown whether many of these microbes in gardens and dumps represents resident members (i.e. not transient) or how uniform community composition is among host ant species. Furthermore, little is known about the compositional changes in microbial community structure between gardens and dumps. In this study, we address whether microbial community structure changes significantly from gardens to dumps. Specifically, we assess if the relative abundance of microbial populations change significantly between these two colony components, and if these changes are similar across colonies.

To address this question, we examine the microbiota in the leaf-cutter ant system using a modified version of microbial membrane lipid analysis, a well-established method for examining broad-scale microbial community structure [27]–[30]. Specifically, using phospholipid fatty acid/fatty acid methyl ester analysis [31], [32] we investigate field-collected and laboratory-maintained colonies of leaf-cutter ants to evaluate the microbial community structure of fungus gardens and refuse dumps, and then use a variety of metrics to compare microbial community structure. Overall, our results indicate that community structure is more similar based on location/substrate (i.e. garden or dump) rather than environment/colony source (lab or field). Analysis of lab colonies indicates strict and consistent community segregation between gardens and dumps. Though our data shows some affect of the lab environment on overall community structure in gardens and dumps, we find this same pattern of segregation reflected in field colonies. Correlation of the detected lipids to their taxonomic designation reveals that in general, gardens contain predominately Gram-negative bacteria. Gram-negative taxa are also present in dumps but our results show a significant enrichment of Gram-positive and anaerobic bacteria. The apparent spatial differences in community structure between leaf-cutter ant gardens and dumps suggest that there is specific partitioning, likely due to substrate availability mediated by ant agricultural practices.

## Materials and Methods

### Sample collection and nomenclature

We sampled gardens and refuse dumps of three species of *Atta* (*A. cephalotes, A. colombica, and A. sexdens*) and four species of *Acromyrmex* (*A. laticeps, A. octospinosus, A. hispidus fallax, A. niger*) leaf-cutter ants. Colonies sampled included, nine laboratory-maintained and five field-collected colonies (Table S1). Lab colonies (L) represented species of both *Atta* and *Acromyrmex* collected in 2003 from sites in Argentina and Panama (Table S1). Lab colonies were maintained at the University of Wisconsin–Madison, housed in 55 L plastic containers consisting of two smaller containers, one for the fungus garden and the other for the refuse dump (Figure 1D). Colonies were fed three times per week using a mixture of oak and maple leaves. Laboratory colonies were first sampled for lipid analysis in 2006 and again in 2008. Field samples were collected from five mature colonies of *Atta colombica* in the Canal Zone of central Panama during a 12-day period from 30 May to 10 June, 2008 (N 09° 07′ W 079° 42′). Most species of leaf-cutter ants excavate deep, subterranean refuse chambers. These chambers are difficult to sample [14], and as such, we chose to focus exclusively on *A. colombica* because refuse material is considerably more accessible. Since mature field colonies forage on a wide variety of host plants, even within a single day [9], it was not possible to detail the type of plant material used by each colony at the time of collection. Two fungus gardens and several dump samples were collected from each colony. Field samples were aseptically collected, stored at −20°C, and subsequently transported on dry ice to our lab at the UW–Madison for lipid analysis. In total, our analysis consisted of 62 samples from four major groups: i) lab gardens (**LG**, sampled in 2006 and 2008), ii) lab dumps (**LD**, sampled in 2006 and 2008), iii) field gardens (**FG**, sampled in 2008), and iv) field dumps (**FD**, sampled in 2008) (Table S1).

### Hybrid PLFA-FAME analysis

Samples of garden and dump were frozen at −20°C, lyophilized, and milled to #40 mesh size prior to phospholipid fatty acid (PLFA) extraction. Membrane lipids were extracted from 1 g lyophilized and milled material in a two-phase aqueous-organic extraction, as described by Bligh and Dyer [33]. Samples were extracted twice and the supernatants were combined after phase separation overnight. The organic phase was isolated and evaporated to dryness using a RapidVap (LabConco, Kansas City, MO). We performed fatty acid methyl ester (FAME) analysis as described by Microbial ID Inc. (MIDI) [34] on the dried organic residue. Lipids were saponified and then subjected to alkaline methanolysis. Lipids were isolated from the mixture in a hexane extraction.

Lipid methyl esters were analyzed in a 2 µL injection using a Hewlett-Packard 6890 Gas Chromatograph configured and maintained for lipid analysis according to the recommendations of MIDI [34]. Gas chromatogram parameters were specified and peaks were identified by the MIDI EUYKARY method (MIDI, Newark, DE). Fatty acid concentration was quantified by comparing peak areas of the samples compared to two internal standards, 9∶0 (nonanoic methyl ester) and 19∶0 (nonadeconoic methyl ester) (Sigma, St. Louis, MO) of known concentration. In all subsequent statistical analyses, we excluded fatty acids that were at an average abundance of <0.5 mol% or present in less than three of the 62 samples.

The following standard fatty acid nomenclature [35], briefly summed up by the following formula, is used throughout this article: A:XωB, where A is the number of carbon atoms in the lipid chain; X is the number of double bonds; B is the position (ω) of the double bond from the methyl end. The prefixes *i* and *a* represent *iso* and *antiso* branching, respectively. The prefix *cy* indicates cyclopropane fatty acid and the suffix 10Me refers to a methyl group on the tenth carbon. The suffixes *c* and *t* represent *cis* or *trans* configuration, respectively [35].

### Community Analysis

#### Richness estimators

**Figure 2:**
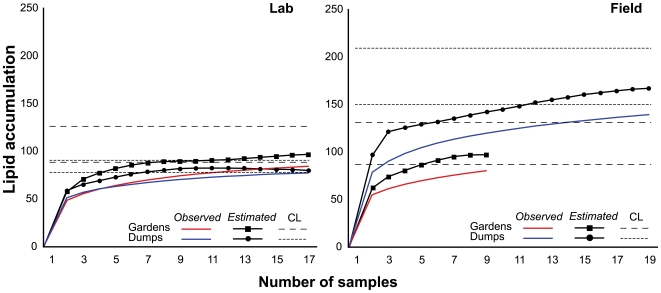
Rarefaction and lipid accumulation curves. Observed and estimated lipid richness of **Lab** colonies (gardens vs. dumps) and **Field** colonies (gardens vs. dumps).

Lipid diversity indices were calculated from the complete dataset using EstimateS v.8.2.0 [36]. We used the *Mao Tau* function to calculate observed richness (accumulation curves) and the *Chao2* function to calculate estimated richness. We used lower and upper *Chao2* log-linear 95% confidence intervals (CI) to create a confidence limit (CL) envelope (Figure 2). Samples were randomized 1000 times without replacement using the biased corrected *Chao2* formula.

#### Analysis of samples using major lipid classes

**Figure 3:**
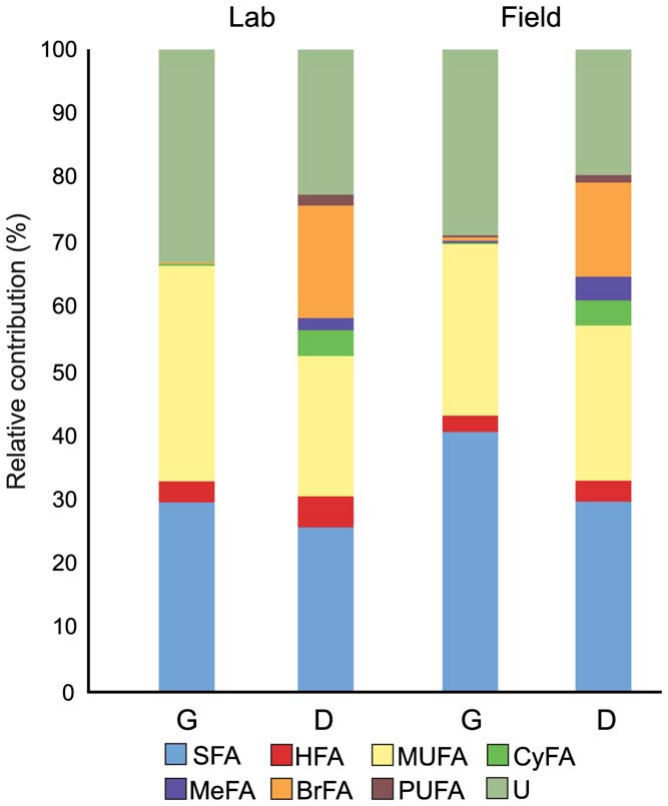
Contribution of major lipid classes to community structure. Chart showing the relative contribution of chemically-related lipid classes to lipid profile of gardens (G) and dumps (D) from lab and field colonies. In addition to ‘Unclassified’ lipids (*U*), gardens predominately contain saturated (*SFA*), monounsaturated (*MUFA*), and hydroxyl (*HFA*) fatty acids. Dumps also contain these lipid classes but are enriched for cyclic (*CyFA*), branched (*BrFA*), methylated (*MeFA*) and polyunsaturated (*PUFA*) fatty acids. These data suggest a broad-scale shift in community structure from fungus gardens to refuse dumps as well as community similarity between respective components of lab and field colonies.

We grouped lipid biomarkers into seven chemically-related classes [37]: saturated fatty acids; hydroxyl fatty acids; monounsaturated fatty acids; cyclic fatty acids; methylated fatty acids; branched fatty acids; and polyunsaturated fatty acids. Certain lipids were too close in size to be separated by gas chromatography, and therefore, an exact identification was not possible. These lipids were grouped together as “Unclassified”. This approach is recognized as an effective means of comparison because major lipid classes correspond to particular groups of organisms [38]. Absolute lipid concentrations (mol%) were averaged by class across sample groups (lab gardens, lab dumps, field gardens, field dumps). Analysis of Variance (ANOVA) and Tukey-Kramer highly significant difference (HSD) tests were performed using the JMP software package v.8.0.1 [39] (Table 1). We also calculated the relative contribution (mol%) of major lipid classes for each sample group (Figure 3).

**Table 1:** Average amount (mol%) of major lipid classes.

*Lipid class* †	*Lab Gardens*	*Lab Dumps*	*Field Gardens*	*Field Dumps*
	n = 17	n = 17	n = 9	n = 19
***SFA***	26.43 (0.80)^b^	22.91 (1.13)^b^	37.09 (3.49)^a^	24.98 (0.97)^b^
	[24.73, 28.13]	[20.53, 25.30]	[29.04, 45.15]	[22.96, 27.01]
***HFA***	2.98 (0.34)^ab^	4.32 (0.62)^a^	2.32 (0.29)^b^	2.68 (0.33)^b^
	[2.26, 3.70]	[3.00, 5.64]	[1.64, 2.99]	[1.99, 3.37]
***MUFA***	29.90 (2.34)^a^	19.41 (1.83)^b^	24.45 (1.50)^ab^	20.31 (1.98)^b^
	[24.94, 34.86]	[15.53, 23.30]	[20.99, 27.92]	[16.16, 24.47]
***BrFA***	0.20 (0.04)^c^	15.56 (1.25)^a^	0.45 (0.10)^c^	12.28 (0.72)^b^
	[0.12, 0.28]	[12.90, 18.22]	[0.23, 0.67]	[10.77, 13.80]
***CyFA***	0.02 (0.02)^b^	3.52 (0.31)^a^	0.17 (0.05)^b^	3.29 (0.41)^a^
	[−0.02, 0.06]	[2.86, 4.17]	[0.06, 0.28]	[2.43, 4.14]
***MeFA***	0.27 (0.06)^c^	1.73 (0.24)^b^	0.31 (0.10)^bc^	3.07 (0.55)^a^
	[0.15, 0.40]	[1.21, 2.24]	[0.08, 0.53]	[1.91, 4.22]
***PUFA***	0.03 (0.01)^b^	1.55 (0.23)^a^	0.30 (0.05)^b^	1.06 (0.16)^a^
	[0.00, 0.06]	[1.06, 2.05]	[0.18, 0.43]	[0.73, 1.39]
**Unclassified** ‡	29.53 (1.53)^a^	20.05 (1.41)^bc^	26.44 (2.01)^ab^	16.33 (1.64)^c^
	[26.29, 32.78]	[17.07, 23.04]	[21.81, 31.07]	[12.88, 19.79]

Values in round brackets are standard error (±S.E). Values in square brackets indicate 95% confidence interval (CI) from Tukey-Kramer HSD analysis.

†Abbreviations: *SFA* (saturated fatty acids); *HFA* (hydroxylated fatty acids); *BrFA* (branched fatty acids); *MUFA* (monounsaturated fatty acids); *CyFA* (cyclic fatty acids); *MeFA* (methylated fatty acids); *PUFA* (polyunsaturated fatty acids).

‡Lipids that could not be classified into one of the seven major classes.

Values not connected by same letter are significantly different (*p*<0.05).

#### Principal component analysis (PCA)

**Figure 4:**
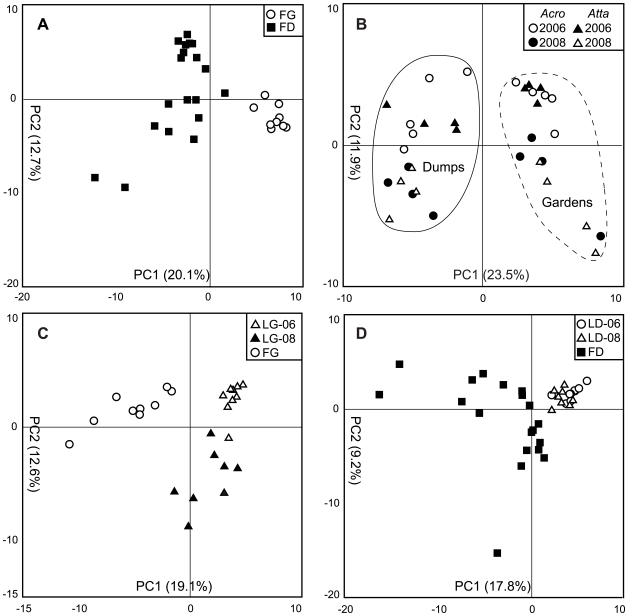
Principal Component Analysis (PCA) of lipid markers recovered from the four sample types. PCA were used to access differences among the four major comparisons explored in this study. Only the first two principal components are shown in all cases (PC1 and PC2). (**A**) Field gardens and field dumps clustered along PC1 however there was considerable variation among dump samples along both principal components. (**B**) From lab samples, clustering along PC1 was related to microhabitat type (garden or dump) and clustering along PC2 was related to year of sampling (2006 or 2008). Solid line indicates dump samples and dashed line indicates garden samples. (**C**) PCA of garden samples showed that variation along PC1 was related to sample source (lab or field). (**D**) PCA of dumps showed a similar pattern in that separation along PC1 was related to sample source. Lab dumps formed a tight cluster likely due in part to the large variation among field samples. **Abbreviations**: LG-06, 2006 lab garden; LG-08, 2008 lab gardens; FG, field garden; LD-06, 2006 lab dumps; LD-08, 2008 lab dumps; FD, field dumps.

For multivariate PCA, all lipids (mol%) were transformed using the square root of the arcsine to approximate normality [40] prior to analysis using the JMP software package v.8.0.1 [39]. We used PCA to explore the following comparisons between the major sample groups: a) lab gardens vs. field gardens; b) lab dumps vs. field dumps; c) lab gardens vs. lab dumps); and d) field gardens vs. field dumps (Figure 4).

#### Cluster analysis of lipid biomarkers

**Figure 5:**
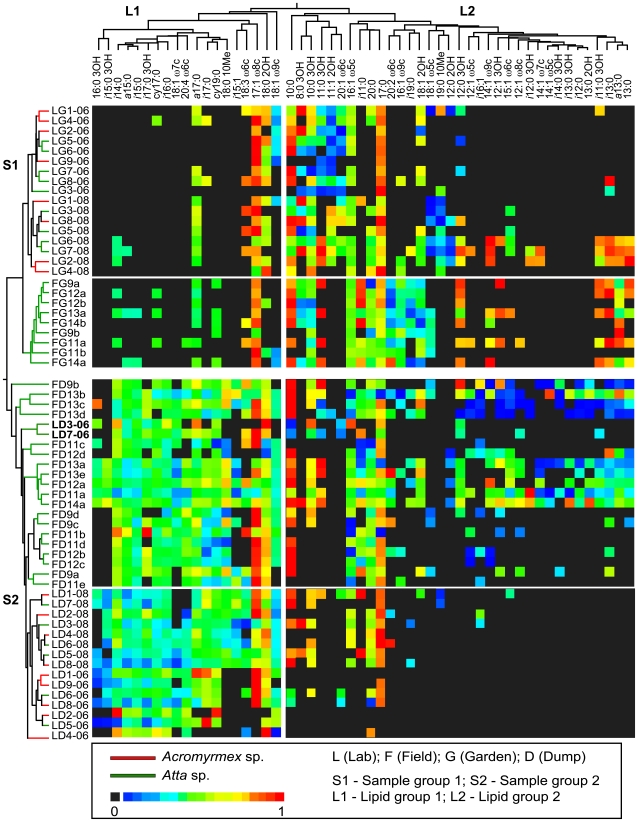
Heat map and dendrograms of lipid distribution. A heat map of samples by lipids. The heat map is arranged according to sample and lipid clustering analysis and mol% values were arcsine-transformed. The dendrogram for the samples (vertical) was calculated using the total number of lipid biomarkers present in each sample. For simplicity, only named lipids were used for the lipid dendrogram (horizontal) and thus for the heat map. Green lines in the vertical dendrogram indicate samples from *Atta* colonies and red lines indicate samples from *Acromyrmex* colonies. Sample codes are as follows: lab (L), field (F), garden (G), and dump (D). Numbers indicate colony identification and the extensions (-06) and (-08) indicate year of sampling. Clustering analysis of samples shows two distinct groups. Group **SI** contains only garden samples and group **SII** contains only dump samples. Within each group, samples generally cluster according to source (lab or field), however two dump samples from lab colonies cluster with the field dumps (LD3-06 and LD7-06 in bold). Overall, field dumps exhibit the highest taxon diversity across lipid groups **LI** and **LII**. Generally, garden communities are well represented by group **LII** taxa and dumps, especially from lab colonies, are dominated by group **LI** taxa. Group **LI** contains primarily branched (*iso* and *antiso*) lipids, indicative of Gram-positive bacteria [38]. Group **LII** is high in hydroxyl lipids and monounsaturated fatty acids, which indicate Gram-negative bacteria [38]. Garden samples, in particular, are enriched with these lipid markers.

Lipid concentrations (mol%) for all samples were compiled into two different profile matrices. In the first profile matrix (the sample matrix), each row corresponded to a sample and each column corresponded to a lipid. In total, we omitted six lipid markers that were common to all samples because they did not affect clustering (9∶0 and 19∶0, which were internal standards and 14∶0, 16∶0, and 18∶0, which are abundant in most organisms [38]). Otherwise, the full lipid dataset was used for clustering analysis. In the profiles second matrix (the lipid matrix), each row corresponded to a lipid and each column corresponded to a sample. Next we used each profile matrix to construct a corresponding similarity matrix by calculating the correlation between all sample pairs and lipid pairs, respectively, using Spearman's rank correlation (with tie-correction), an approach that has been used to effectively cluster a variety of genetic datasets [41]–[43]. We then used each similarity matrix to generate corresponding dendrograms using the neighbor-joining program in PHYLIP [44] and visualized with the Phylodendron phylogenetic tree printing webserver (http://iubio.bio.indiana.edu/treeapp/treeprint-form.html, accessed: 11/09/2009). Thus, we created a sample dendrogram (*S*) and a lipids dendrogram (*L*). We then reorganized the original sample profile matrix to reflect the topology of each dendrogram, samples on the horizontal axis and lipids on the vertical axis. In order to approximate normality, mol% concentrations were transformed using the square root of the arcsine [40] and each raw arcsine transformed data point was represented in a heat map using the Microsoft Excel Yabs Heat map macro (http://homepage.mac.com/yabyab/program/heatmap.html, accessed 11/09/2009) (Figure 5).

#### Correlation of lipids to microbial taxonomy

To determine the set of common microbial taxa within each of the four major groups (**LG**, **FG**, **LD**, and **FD**) we designed a lipid-to-microbial map. This allowed us to identify lipids common to samples within groups and then correlate each lipid to known microbial classification. Using previously published reports [28], [32], [38], [45]–[54] we first created a lipid-microbe classification table to tabulate each sample's complement of microbial taxa. Only lipids corresponding to reported taxa were used in the analysis. In total, we identified 31 lipids from our full dataset that corresponded to reported taxa. The remaining lipid markers had no known identity and thus could not be considered in this particular analysis. For each lipid, we calculated average mol% concentration (±S.E.) within groups. Finally, we used ANOVA and Tukey's HSD to assess differences in microbial taxa between major groups (**LG**, **FG**, **LD**, and **FD**). In some cases, individual lipids corresponded to more than one group of organisms. In these instances, all taxonomic identifications were considered in the analysis, and the first one listed occurred most often in the literature (Table 2).

**Table 2:** Indicator lipids from lab (2008) and field colonies†.

Lipid	Lab Gardens	Lab Dumps	Field Gardens	Field Dumps	Indicators‡
**8∶0-3OH**	0.12(0.05)^ab^	ND§ ^bc^	0.13(0.04)^a^	ND^c^	Gm-
**10∶0-3OH**	0.59(0.17)^a^	0.15(0.04)^a^	0.87(0.16)^b^	0.11(0.03)^b^	Gm-, γ-proteo (*EV*)
**16∶0-2OH**	0.58(0.04)^ab^	0.93(0.07)^a^	0.41(0.05)^b^	0.70(0.09)^ab^	Gm-, *CFB*
**16∶0-3OH**	ND^b^	0.20(0.08)^a^	ND^b^	0.08(0.03)^ab^	Gm-, γ-proteo (*XP*), *CFB*
**18∶0-2OH**	0.51(0.13)^ab^	0.76(0.06)^ab^	0.25(0.17)^b^	0.80(0.13)^a^	Gm-, Fun
**18∶1-2OH**	0.14(0.05)^b^	0.05(0.05)^b^	0.55(0.09)^a^	0.11(0.03)^b^	Gm-
***i*** **11∶0**	ND^b^	0.02(0.01)^b^	0.09(0.02)^ab^	0.22(0.04)^a^	Gm+
***a*** **13∶0**	0.02(0.01)^ab^	ND^b^	0.07(0.01)^ab^	0.08(0.02)^a^	Gm+, *BC*, Act
***i*** **13∶0**	0.02(0.01)^b^	ND^b^	0.02(0.01)^b^	0.16(0.04)^a^	Gm+, *BC*
***i*** **14∶0**	ND^b^	1.07(0.13)^a^	ND^b^	0.70(0.10)^a^	Gm+, *BC*, Act
***a*** **15∶0**	ND^c^	4.25(0.47)^a^	ND^c^	1.86(0.24)^b^	Gm+, *BC*, Act
***i*** **15∶0**	ND^c^	4.38(0.28)^a^	0.03(0.02)^c^	3.41(0.27)^b^	Gm+, *BC*, Act
***i*** **15∶1**	ND^b^	0.32(0.05)^a^	ND^b^	0.19(0.05)^a^	Gm+
***i*** **16∶0**	ND^c^	3.30(0.28)^a^	ND^c^	2.28(0.21)^b^	Gm+, *BC*, Act
***a*** **17∶0**	0.14(0.02)^b^	1.22(0.09)^a^	0.06(0.02)^b^	1.00(0.08)^a^	Gm+, *BC*
***i*** **17∶0**	ND^b^	0.81(0.10)^a^	ND^b^	1.02(0.14)^a^	Gm+, *BC*
***i*** **19∶0**	ND^b^	0.05(0.03)^ab^	0.14(0.03)^a^	0.04(0.01)^b^	Gm+, *BC*
***i*** **15∶0-3OH**	ND^b^	0.53(0.10)^a^	ND^b^	0.05(0.02)^b^	Gm-, γ-proteo (*XP*), *CFB*
***i*** **17∶0-3OH**	ND^b^	1.11(0.20)^a^	ND^b^	0.31(0.08)^b^	Gm-, *CFB*, *BC*
**16∶1ω5c**	0.28(0.05)^b^	0.28(0.07)^b^	0.43(0.07)^b^	1.92(0.42)^a^	Fun,Gm-,γ-proteo(*XP*), *CFB*,*BC*
**16∶1ω7c**	0.62(0.11)^b^	4.12(0.44)^a^	0.30(0.04)^b^	3.79(0.64)^a^	Gm-, *BC*, Anae
**17∶1ω8c**	0.61(0.06)^a^	0.73(0.06)^a^	0.33(0.03)^b^	0.54(0.06)^ab^	Gm-
**18∶1ω5c**	9.87(3.64)^a^	ND^b^	1.31(0.53)^b^	1.77(0.77)^b^	Gm-
**18∶1ω7c**	ND^b^	1.94(0.83)^a^	ND^b^	1.94(0.32)^a^	Gm-, γ-proteo (*EV*), *BC*
**18∶1ω9c**	1.70(1.70)^b^	13.27(1.30)^a^	3.89(2.58)^b^	4.94(0.59)^b^	Fun, Gm+,Gm-(anae),*BC*,Act
***cy*** **17∶0**	ND^c^	2.29(0.16)^a^	0.06(0.03)^c^	1.28(0.17)^b^	Gm-(anae),γ-proteo,*BC*
***cy*** **19∶0**	ND^b^	1.61(0.16)^a^	0.11(0.05)^b^	1.86(0.31)^a^	Gm-(anae),Gm+,γ-proteo,*BC*
***cy*** **2OH19∶0**	ND^b^	0.03(0.02)^ab^	ND^b^	0.15(0.04)^a^	Gm-(anae), Gm-
**10Me18∶0**	ND^b^	0.51(0.06)^a^	ND^b^	0.48(0.09)^a^	Act
**10Me19∶0**	0.26(0.05)^a^	0.09(0.06)^b^	ND^b^	ND^b^	Act
**20∶2ω6c**	ND^b^	0.05(0.04)^b^	0.27(0.04)^a^	0.07(0.02)^b^	*BC*

Table of named lipids found in a majority of samples from lab gardens and refuse dumps. Values are given as mol% (S.E).

†After [28], [32], [38], [45]–[54].

‡Abbreviations: Gram-negative (Gm-); Gram-positive (Gm+); γ-Proteobacteria (γ-proteo−XP = *Xanthomonas/Pseudomonas; EV* = *Enterobacter/Vibrio*); *Cytophaga-Flexibacter-Bacteroides* group (CFB); *Bacillus-Clostridium* group (BC); Actinobacteria (Act); Fungi (Fun), Anaerobic (anae).

§ND indicates lipid was Not Detected.

Values not connected by same letter are significantly different (*p*<0.05).

## Results

We determined the structure of microbial communities in leaf-cutter ant gardens and dumps. Our sampling of gardens (n = 17) and dumps (n = 17) from lab-maintained colonies and field-collected gardens (n = 9) and dumps (n = 19) from five mature colonies of *Atta colombica* resulted in a complete dataset that consisted of 156 lipid markers (Table S2).

### Lipid richness and abundance

#### Lipid richness

We calculated lipid richness for lab gardens, lab dumps, field gardens, and field dumps (Figure 2). Richness analysis indicated that gardens were well sampled, and that richness was similar between lab and field fungus gardens. Lab dumps were also sufficiently sampled; however, field dumps were significantly richer than all other groups and expressed unsampled diversity.

#### Abundance of major lipid classes

We separated lipids into the following seven chemically-related classes and calculated the average mol percentage (mol%) contribution of each class: saturated fatty acids, monounsaturated fatty acids, hydroxy fatty acids, cyclic fatty acids, methylated fatty acids, branched fatty acids and polyunsaturated fatty acids. Lab and field gardens were composed almost entirely of saturated and monounsaturated fatty acids. Hydroxy fatty acids, though not as abundant, constituted the third largest lipid class detected in the gardens (Figure 3). Dumps contained these three classes, but were also enriched for cyclic fatty acids, methylated fatty acids, branched fatty acids, and polyunsaturated fatty acids (Figure 3). One-way ANOVA revealed that each of the seven major lipid classes differed significantly across the four sample groups: saturated fatty acids, *F*(3,58) = 14.33, *p*<0.001; monounsaturated fatty acids, *F*(3,58) = 5.84, *p*<0.001; hydroxy fatty acids, *F* (3,58) = 3.67, *p* = 0.0172; cyclic fatty acids, F (3,58) = 39.06, *p*<0.001; methylated fatty acids, *F*(3,58) = 13.74, *p* = 0.0015; branched fatty acids; *F*(3,58) = 91.27, *p*<0.001; polyunsaturated fatty acids, *F*(3,58) = 18.71, *p*<0.001.

Tukey-Kramer post-hoc comparisons revealed that gardens from lab and field colonies differed only in the amount of saturated fatty acids present (*p*<0.001) (Table 1), and there was no significant difference in the amount of other six lipid classes (Table 1). Lab dumps contained greater amounts of hydroxy fatty acids (*p* = 0.035) and branched fatty acids (*p* = 0.018) than that of field dumps, but lower amounts of methylated fatty acids (*p* = 0.036). Lab and field dumps did not differ in the amounts of saturated fatty acids, monounsaturated fatty acids, cyclic fatty acids, and polyunsaturated fatty acids (Table 1).

We found no difference in the amounts of saturated fatty acids and hydroxy fatty acids between lab gardens and lab dumps (Table 1). Lab dumps had significantly greater amounts of branched fatty acids (*p*<0.001), methylated fatty acids (*p* = 0.024), cyclic fatty acids (*p*<0.001), and polyunsaturated fatty acids (*p*<0.001) than lab gardens. Lab gardens contained significantly greater amounts of monounsaturated fatty acids compared to lab dumps (*p* = 0.0024). Field dumps contained significantly greater amounts of branched fatty acids (*p*<0.001), methylated fatty acids (*p*<0.001), cyclic fatty acids (*p*<0.001), and polyunsaturated fatty acids (*p* = 0.025) than that of field gardens (Table 1). Field gardens had significantly greater amounts of saturated fatty acids than dumps (*p*<0.001). We found no differences in the amount of monounsaturated fatty acids or hydroxy fatty acids levels (Table 1).

### Principal component analysis

We used Principal Component Analysis (PCA) to assess similarity in microbial community structure across all samples (Figure 4). In total, we performed four different PCAs representing the four main comparisons used throughout this study. PCA of field samples revealed garden samples clustered to the exclusion of dump samples separated primarily along Principal Component 1 (PC1) (Figure 4A). Dump samples again exhibited considerable variation and no clustering based on host ant colony was observed (data not shown). PC1 accounted for 20.1% of the observed variation and PC2 accounted for 12.7% of the variation. Lab samples exhibited a similar pattern in that gardens and dumps formed distinct clusters (Figure 4B). Based on the separation of samples along PC1, clustering was related to the source of the sample (either garden or dump) and separation of samples along PC2 was related to year of sampling (2006 or 2008). PC1 accounted for 23.5% of the observed variation and PC2 accounted for 11.9% of the variation. PCA of garden samples from lab and field colonies indicated that clustering along PC1 was explained by sample source (lab or field) (Figure 4C). Lab samples clustered according to year sampled along PC2. PC1 accounted for 19.1% of the variation and PC2 accounted for 12.6% of the variation. Examination of field samples revealed considerable variation between samples and no clustering was evident based on nest or location origin (data not shown). Dump samples from lab colonies formed a single, tight cluster from both 2006 and 2008 (Figure 4D) likely due to the large variation of field samples. PC1 accounted for 17.8% of the variation and PC2 accounted for 9.2% of the variation.

### Heat map and clustering analysis

We performed heat map and clustering analysis using a subset of named lipid markers in our dataset to help explain the observed patterns from our richness estimators, major lipid class analysis, and PCA. Lipids formed two main clusters within the lipid (*L*) analysis, groups *L1* and *L2*, as shown in Figure 5. Furthermore, we found that gardens and dumps also formed individual clusters within the sample (*S*) dendrogram. Group S1 was composed entirely of garden samples (from lab and field colonies) and sample group S2 contained only lab and field dump samples. Thus, sample clustering was related to microhabitat type (gardens or dumps) and not sample source (lab or field). However, sub-clustering was apparent within each major group, with lab samples forming their own groups distinct from field samples.

Further analysis revealed that gardens were typically composed of group *L2* lipids (specifically hydroxy fatty acids: 8∶0-3OH; 10∶0-3OH; 11∶0-3OH; 18∶0-2OH; and monounsaturated fatty acids: 16∶1ω5c; 17∶1ω8c; 18∶1ω9c) and contained very few lipid markers from group *L1*. In contrast, dumps were enriched for group *L1* lipids and clustered based primarily on branched (*i*14∶0, *a*15∶0, *i*15∶0, *i*15∶1, *i*16∶0, *i*17∶0, *a*17∶0, *i*17∶0-3OH) and cyclic (*cy*17∶0 and *cy*19∶0) fatty acids. Branched fatty acids were absent from garden samples (with the exception of *a*17∶0, present in 18 of 26 samples). Cyclic fatty acids were present in less than half of the field garden samples and only one lab garden sample. Cluster analysis revealed no grouping of lab samples by ant genus (neither gardens nor dumps), or field samples according to source nest. There was a major shift from group *L2* lipids in lab gardens to group *L1* lipids in lab dumps. *L2* lipids were very rare in lab dumps.

### Correlation of lipid markers to microbial taxonomy

We analyzed 31 individual lipids across lab gardens, lab dumps, field gardens, and field dumps in order to determine which lipids were enriched between samples. Table 2 summarizes these results.

#### Analysis of taxa between garden samples

Ten of the 31 (32%) markers were not found in either lab or field gardens, while only 7 of 31 markers tested (23%) were found in significantly different amounts between lab and field gardens. Three were found to be present in greater amounts in lab gardens than in field gardens: 2 lipids corresponding to Gram-negative taxa (17∶1ω8c and 18∶1ω5c) and 1 actinomycete marker (19∶0 10Me). Four lipids were found to be present in greater amounts in field gardens than in lab gardens, including a general Gram-negative marker (18∶1-2OH), a marker specific to γ-Proteobacteria (*Enterobacter/Vibrio*) (10∶0-3OH) and 2 Gram-positive markers, specifically corresponding to bacteria in the phylum Firmicutes (*Bacillus, Lacobacillus*, and *Enterococcus*; *i*19∶0 and 20∶2ω6c).

#### Analysis of taxa between dump samples

Comparison of dump samples from lab and field colonies revealed that 12 of 31 (39%) markers were detected in significantly different amounts. Of these, 8 (26%) markers were found in greater amounts in lab dumps than in field dumps: 3 Gram-negative markers; 10∶0-3OH, *i*15∶-3OH, and *i*17∶0-3OH, corresponding to *Enterobacter-Vibrio*, *Xanthomonas-Pseudomonas*, and *Cytophaga-Flexibacter-Bacteroides*, respectively. Additionally, 3 markers were specific to the *Bacillus-Clostridium* group (18∶1ω9c, *i*15∶0, and *i*16∶0), 1 to general Gram-positive (*a*15∶0), and 1 to Gram-negative anaerobes (*cy*17∶0). In total, 4 markers (13%) were found in greater amounts in field dumps than in lab dumps. These include 3 markers indicating the presence of Gram-positive taxa, specifically the *Bacillus-Clostridium* group (*BC*) (*i*11∶0, *a*13∶0, *i*13∶0) and 1 Gram-negative marker (16∶1ω5c). In general, lab dumps contained higher levels of markers indicative of both Gram-negative and Gram-positive bacteria.

#### Analysis of taxa within lab colonies

Of the 31 lipids analyzed, 18 (58%) were detected in significantly different (*p*<0.05) amounts in gardens compared to refuse dumps (Table 2). Of these, only 2 (11%) lipids were encountered in greater amounts in the gardens and 16 (89%) were present in greater amounts in the dumps. Garden samples contained greater amounts of 18∶1ω5c and 19∶0 10Me, which correspond to Gram-negative bacteria and Actinobacteria, respectively. We did not detect lipid 18∶1ω5c in any dump samples. Of the 16 lipids present in greater amounts from dumps samples, 8 (50%) correspond to Gram-positive taxa and 2 (13%) lipids indicated the presence of Gram-negative anaerobes (*cy*17∶0 and *cy*19∶0). Lab dumps were also enriched with a different type of actinomycete marker (18∶0 10Me) as well as 5 markers indicative of Gram-negative bacteria. Of the 16 markers detected in greater amount from dump samples, 12 (75%) were not detected in garden samples.

#### Analysis of taxa within field colonies

Analysis of field samples revealed similar patterns to lab colonies. Of the 31 lipids analyzed, 20 (65%) were found in significantly different amounts between field garden and dumps. Of these, 3 (15%) were present in greater amounts in fungus gardens and 17 (85%) were higher in refuse dumps (7 of which were not detected in garden samples). Similar to lab dumps, field dumps are enriched for Gram-positive bacteria. In fact, 9 of the markers present in greater amounts from dumps correspond to Gram-positive taxa including members of the Firmicutes (*Bacillus-Clostridium*). Again, as seen in lab dumps, field dumps were enriched for lipids indicating the presence of Gram-negative anaerobes (*cy*17∶0 and *cy*19∶0).

## Discussion

In this study, we present the first broad-scale analysis of microbial community structure within, and across, leaf-cutter ant colonies using microbial lipid analysis. Membrane lipids, essential components of living cells, provide a reliable and quantitative assessment of general microbial community structure [29], [30], [38]. Furthermore, because lipids exhibit structural diversity that can be linked to specific microbial taxa, community assemblages can be resolved, to some degree, at a broad phylogenetic scale [32], [54], [55]. Perhaps the biggest strength of a lipid-based approach, as compared to other microbial community assays, is that because phospholipid fatty acids are not found in storage molecules and degrade rapidly during cell death, it provides a census of the current living community (i.e., it is a proxy for living biomass) [28]. Many genetic-based approaches contain inherent biases such as unequal efficiency in DNA extraction, preferential amplification of certain taxa, and overrepresentation of particular groups in public databases [29], [56]. Further, genetic approaches reveal total diversity and say little about expressed diversity. Because diversity in many systems is sizable, genetic approaches may fail to identify fine-scale treatment effects [28]. Thus, lipid membrane analysis provides a logical first step for detecting shifts in community structure and establishes a framework for more intensive analyses of community composition.

Using microbial lipid analysis and various community metrics, we demonstrate that the fungus gardens and refuse dumps of leaf-cutter ants are composed of a diverse, yet distinct, community of microbes. In all colonies surveyed, the same pattern of broad-scale community change and segregation was apparent. In general, gardens contained a high abundance of Gram-negative bacteria, specifically markers indicative of Proteobacteria and Bacteroidetes. Though there was some affect of colony source (i.e. lab vs. field) on sample relatedness, we found striking similarities among community structure of lab-maintained and field-collected gardens, indicating the possibility that spatiotemporally stable communities are consistently maintained over time. Thus, our findings represent important support of other recent work [26] specifically indicating that the fungus gardens of leaf-cutter ants contain additional microbial symbionts to the fungal cultivar.

In contrast to gardens, dumps were found to be more diverse and enriched for Gram-positive and anaerobic bacteria. In addition, field dumps contained considerably richer communities than lab dumps and our results indicated the presence of a commonly-associated community across all samples, even though there was some affect of colony source on sample relatedness. This study is among the first to assess the microbial composition of leaf-cutter ant refuse dumps.

### Community structure of leaf-cutter ant colonies

Our findings that microbial communities differ markedly between gardens and dumps are consistent across colonies (in both lab and field), host genera (*Atta* and *Acromyrmex*; lab only), and time (2006 vs. 2008; lab only). Our analysis of field colonies indicates significant shifts in community structure between gardens and dumps (Figure 2, 3, 4A, and 5, Table 1 and 2). Given that refuse dumps of *Atta colombica* are exposed to numerous environmental inputs while field gardens remain relatively sheltered from the environment in subterranean chambers, these results are perhaps not too surprising. However, our data reveal similar patterns in lab colonies. Here too, large-scale and consistent community changes are evident between gardens and dumps (Figure 3, 4B, and 5, Table 1 and 2). Many of the environmental variables inherent to field colonies are likely greatly reduced, or completely absent, in the laboratory. Moreover, gardens and dumps in the lab are housed in close proximity within the same container (Figure 1D), further minimizing any potential effects of spatial separation.

Specifically, we find that microbial communities of fungus gardens, whether from lab or field colonies, are more similar to each other than to their corresponding refuse dumps. Diversity indices reveal no difference in taxa richness between lab and field gardens (Figure 2). Relatedness among samples reflects the source of the host colony (lab or field; Figure 4C and 5); however, our finding that Gram-negative bacteria dominate gardens is consistent across lab and field colonies. Direct analysis of lipids common between gardens reveal the presence a number of groups including those belonging to γ-Proteobacteria and Bacteroidetes (Table 2). Overall, the similarities between lab and field gardens are unexpected given that they are exposed to dramatically different environmental conditions (and hence potential microbial colonizers). For example, lab gardens are maintained in relatively sterile containers and are fed a very limited diet. In contrast, field gardens reside in close quarters to the surrounding soil and colonies forage on a wide variety of plant species [9]. A strict lab diet would likely contribute similar microbial inputs whereas a varied diet potentially contains a much greater diversity of microorganisms [57] and thus represents a larger source of possible colonizers. This finding provides evidence for strong substrate control over bacterial community structure, in contrast to the more commonly assumed environmental control.

We also demonstrated that lab dumps share many community members with field dumps. This is in spite of the fact that a) taxa diversity appeared much more limited in the lab environment and b) field dumps exhibited considerable variation between samples (Figure 2 and 4D). As is in the case of fungus gardens, relatedness of microbial communities in refuse dumps generally reflected sample source (Figure 4D and 5). In the lab, refuse dumps are maintained in relatively sterile conditions and have no exposure to external environmental variables. Conversely, field dumps are exposed to continual environmental inputs from rain, soil, falling debris, and animal and plant incursions [15]. It is possible that many members of field dump microbial communities are recruited from outside the ant system via some combination of these factors. Alternatively, it is plausible that the dump community is specialized, and that refuse dumps of other colonies act as a source of microbial colonizers via a yet unknown mechanism. Regardless, while lab dump diversity levels seemed to be hindered by limited environmental inputs, our data indicated that lab and field dumps share many community characteristics. For example, phylogenetic analysis of select markers in these dumps revealed that the majority of taxa (61%) were present in equal concentrations (Table 2). Unlike fungus gardens, both lab and field dumps were enriched for Gram-positive (specifically in the *Bacillus-Clostridium* group) and anaerobic bacteria (Table 2). In fact, many taxa present in refuse dumps were not detected in fungus gardens (Figure 3 and 5, Table 2). Since garden waste material is the main source of microbial inoculum for dumps (especially in lab colonies), this indicated that certain organisms pass through the fungus garden and flourished in the dump.

Although we did not test the effect of host ant taxa on microbial community structure using field samples, our analyses of lab colonies revealed no effect of host ant genus (*Atta* vs. *Acromyrmex*) on microbial community structure in either gardens or dumps (Figure 4B and 5). Though *Atta* and *Acromyrmex* are known to cultivate genetically similar fungal cultivars [58], [59], many aspects of their natural history are fundamentally different (e.g. colony size, degree of worker polymorphism, habitat preference, forage material, etc) [3]. Additionally, although our data showed some effect of sample year on community structure in lab colonies (Figure 4B and 5), the same pattern of partitioning was evident (Figure 5). Overall, we found that gardens and dumps retained their community structure across ant genera and time (2006 and 2008), with Gram-negative bacteria dominating in the gardens and Gram-positive bacteria abundant in the dumps. This pattern strongly suggests that these assemblages can be strictly maintained.

### Leaf-cutter Ant Colonies as Natural Composts

In general, our data suggest that gardens are dominated by Gram-negative bacteria and dumps by Gram-positive bacteria. Given that community structure seems largely independent of external environmental variables (temperature, humidity, rainfall, etc.), host ant taxa, and spatiotemporal factors, we propose an alternative explanation for this pattern of community partitioning. As we suggest above, leaf-cutter ant colonies are a two-stage plant biomass degradation system. This is analogous to human composting activities, a process whereby a complex community of microorganisms under controlled conditions degrades organic material [60]. Microbial community succession in composting systems is coupled with the degradation of plant cell wall polysaccharides (hemicellulose, cellulose, and lignin) [61]. Using an agricultural waste compositing system, Yu *et al.* [61] found that early stages of composting were marked by hemicellulose and cellulose degradation and dominated by Gram-negative bacteria, specifically Proteobacteria [61]. Later stages of composting were characterized by increased lignin degradation coupled with shifts in microbial communities to more Gram-positive taxa such as Actinobacteria. In a study of the fungus garden microbiome of *Atta colombica* leaf-cutter ants, Suen *et al.* [13] calculated the amount of plant polysaccharides in the top (input) and bottom (output) of fungus gardens. The authors determined that hemicellulose and cellulose degradation occurred through the fungus garden while lignin content remained relatively unchanged. Thus, garden waste (input for the refuse dump) had higher proportions of lignin and lower proportions of hemicelluloses and cellulose, relative to fungus garden plant biomass. Additionally, they demonstrated the abundance and dominance of Gram-negative bacteria in the fungus garden, specifically those belonging to the phyla Proteobacteria and Bacteroidetes, which confirms results presented in this study.

The microbial composition data presented in this study, together with previous reports of plant polysaccharide degradation through these colonies, provides strong evidence that leaf-cutter ant colonies may play a role as natural composts in tropical ecosystems. The fungus garden may thus serve as the initial site for early-stage composting while the dump serves as a concentrated area of late-stage composting. These processes are strictly maintained by the fungicultural behavior of the ants: plant biomass degraded by the fungal garden microbiota are removed and deposited into refuse dumps. This segregating behavior could thus act as a mechanism for the observed microbial structure in the fungus gardens and dumps of leaf-cutter ant colonies. By partitioning substrate in this manner, these ants likely promote the concentration and proliferation of specific microbial members throughout the different components of their colonies. This segregating behavior is potentially advantageous for the ants, as the strict maintenance of a specific microbial community in the garden may promote the stable maintenance of the fungus garden, while increasing the growth of their mutualistic fungus, thereby increasing food production. While in the dumps, greater diversity may increase the efficiency of waste degradation, and require less behavioral intervention by the ants. Our study of the microbial communities associated with leaf-cutter ant colonies provides insight into how host behavior can potentially influence the composition of microbial assemblages, and further illustrates the important role of symbiotic associations in shaping the dynamics of these communities.

## Supporting Information

Table S1 Collection information.
(0.13 MB DOC)

Table S2 Lipid quantites (mol%) found in each sample.
(0.20 MB PDF)
