# Supplementary material for: Microbial Community Structure of Leaf-Cutter Ant Fungus Gardens and Refuse Dumps
Source: PLoS One. 2010 Mar 29;5(3):e9922. doi: 10.1371/journal.pone.0009922 (PMC2847949; doi:10.1371/journal.pone.0009922)
Supplement: Table S1 — Collection information. (0.13 MB DOC) [file pone.0009922.s001.doc]

Supplementary Table 1. Collection information

| **Colony ID** | **Host ant** | **Origin** | **Source** | **Habitat** | **Yr^†^** | **Sample Code*** |
| --- | --- | --- | --- | --- | --- | --- |
| **UGM030327-02** | *Acromyrmex hispidus fallax* | Argentina | Lab | Garden | 2006 | LG1-06 |
|  |  |  |  |  | 2008 | LG1-08 |
|  |  |  |  | Dump | 2006 | LD1-06 |
|  |  |  |  |  | 2008 | LD1-08 |
| **UGM030330-04** | *Acromyrmex laticeps* | Argentina | Lab | Garden | 2006 | LG2-06 |
|  |  |  |  |  | 2008 | LG2-08 |
|  |  |  |  | Dump | 2006 | LD2-06 |
|  |  |  |  |  | 2008 | LD2-08 |
| **UGM030330-07** | *Atta sexdens* | Argentina | Lab | Garden | 2006 | LG3-06 |
|  |  |  |  |  | 2008 | LG3-08 |
|  |  |  |  | Dump | 2006 | LD3-06 |
|  |  |  |  |  | 2008 | LD3-08 |
| **CC030403-09** | *Acromyrmex octospinosus* | Argentina | Lab | Garden | 2006 | LG4-06 |
|  |  |  |  |  | 2008 | LG4-08 |
|  |  |  |  | Dump | 2006 | LD4-06 |
|  |  |  |  |  | 2008 | LD4-08 |
| **CC031208-01** | *Atta colombica* | Panama | Lab | Garden | 2006 | LG5-06 |
|  |  |  |  |  | 2008 | LG5-08 |
|  |  |  |  | Dump | 2006 | LD5-06 |
|  |  |  |  |  | 2008 | LD5-08 |
| **CC030329-01** | *Atta sexdens* | Argentina | Lab | Garden | 2006 | LG6-06 |
|  |  |  |  |  | 2008 | LG6-08 |
|  |  |  |  | Dump | 2006 | LD6-06 |
|  |  |  |  |  | 2008 | LD6-08 |
| **A. CEPH 6** | *Atta cephalotes* | Panama | Lab | Garden | 2006 | LG7-06 |
|  |  |  |  |  | 2008 | LG7-08 |
|  |  |  |  | Dump | 2006 | LD7-06 |
|  |  |  |  |  | 2008 | LD7-08 |
| **UGM030327-03** | *Acromyrmex niger* | Argentina | Lab | Garden | 2006 | LG8-06 |
|  |  |  |  |  | 2008 | LG8-08 |
|  |  |  |  | Dump | 2006 | LD8-06 |
|  |  |  |  |  | 2008 | LD8-08 |
| **SP030327-01** | *Acromyrmex hispidus fallax* | Argentina | Lab | Garden | 2006 | LG9-06 |
|  |  |  |  | Dump | 2006 | LD9-06 |
| **JS080530-09** | *Atta colombica* | Panama | Field | Garden | 2008 | FG9a |
|  |  |  |  |  | 2008 | FG9b |
|  |  |  |  | Dump | 2008 | FD9a |
|  |  |  |  |  | 2008 | FD9b |
|  |  |  |  |  | 2008 | FD9c |
|  |  |  |  |  | 2008 | FD9d |
| **JS080531-11** | *Atta colombica* | Panama | Field | Garden | 2008 | FG11a |
|  |  |  |  |  | 2008 | FG11b |
|  |  |  |  | Dump | 2008 | FD11a |
|  |  |  |  |  | 2008 | FD11b |
|  |  |  |  |  | 2008 | FD11c |
|  |  |  |  |  | 2008 | FD11d |
|  |  |  |  |  | 2008 | FD11e |

Supplementary Table 1 continued

| **Colony ID** | **Host ant** | **Origin** | **Source** | **Habitat** | **Yr^†^** | **Sample Code*** |
| --- | --- | --- | --- | --- | --- | --- |
| **JS080607-12** | *Atta colombica* | Panama | Field | Garden | 2008 | FG12a |
|  |  |  |  |  | 2008 | FG12b |
|  |  |  |  | Dump | 2008 | FD12a |
|  |  |  |  |  | 2008 | FD12b |
|  |  |  |  |  | 2008 | FD12c |
|  |  |  |  |  | 2008 | FD12d |
| **JS080608-13** | *Atta colombica* | Panama | Field | Garden | 2008 | FG13a |
|  |  |  |  | Dump | 2008 | FD13a |
|  |  |  |  |  | 2008 | FD13b |
|  |  |  |  |  | 2008 | FD13c |
|  |  |  |  |  | 2008 | FD13d |
|  |  |  |  |  | 2008 | FD13e |
| **JS080610-14** | *Atta colombica* | Panama | Field | Garden | 2008 | FG14a |
|  |  |  |  |  | 2008 | FG14b |
|  |  |  |  | Dump | 2008 | FD14a |

† Indicates the year of lipid extraction. For collection year, all lab colonies were collected in 2003 and all field colonies in 2008.

*Sample code: We use the following scheme for sample naming throughout the manuscript: XY#, where X is the **Source** of the sample (**L**ab or **F**ield), Y is the **System Component** (**G**arden or **D**ump) and # is a number indicating the colony identification. In the case of lab colonies, the extensions **-06** and **-08** indicate the year of sampling
