# Supplementary material for: Microbial Community Structure of Leaf-Cutter Ant Fungus Gardens and Refuse Dumps
Source: PLoS One. 2010 Mar 29;5(3):e9922. doi: 10.1371/journal.pone.0009922 (PMC2847949; doi:10.1371/journal.pone.0009922)
Supplement: Table S2 — Lipid quantites (mol%) found in each sample. (0.20 MB PDF) [file pone.0009922.s002.pdf]

Supplementary Table 2: Lipid quantites (mol%) found in each sample.

Empty cells indicate lipid was not detected in that sample. Unk = Unknown; SiF = Sum in Feature

Sample Code	System Component	Source	8:0 3OH	9:0	9:0 3OH	10:0	10:0 3OH	*i*10:0	11:0	11:0 2OH	11:0 3OH	11:0 DiMethyl Acetal	*i*11:0	*i*11:0 3OH	11:1 2OH	12:0	12:0 2OH	12:0 3OH
LG1-08	Garden	Lab	0.026	0.069		0.476	0.152		0.039				0.004			0.888		
LG2-08	Garden	Lab		0.034		0.128	0.334				0.027			0.084	0.142	0.482	0.071	0.088
LG3-08	Garden	Lab	0.221	0.111		0.408	0.891								0.097	0.786		
LG4-08	Garden	Lab		0.042		0.345	0.150									0.553		
LG5-08	Garden	Lab	0.440	0.092		0.235	1.535		0.022						0.174	0.593		
LG6-08	Garden	Lab	0.123	0.074		0.280	0.857		0.031		0.020			0.011	0.189	0.549	0.091	0.106
LG7-08	Garden	Lab	0.035	0.076		0.374	0.185				0.028			0.059	0.085	0.712	0.115	0.039
LG8-08	Garden	Lab	0.075	0.058		0.243	0.576								0.161	0.425	0.065	0.137
LD1-08	Dump	Lab		0.046		0.232	0.149	0.012	0.025		0.103		0.078			0.546		
LD2-08	Dump	Lab		0.023												0.306		
LD3-08	Dump	Lab	0.040	0.143		0.206	0.206	0.029								0.585		
LD4-08	Dump	Lab		0.053												0.665		
LD5-08	Dump	Lab	0.062	0.155		0.240	0.372						0.046			0.551		
LD6-08	Dump	Lab		0.069			0.159									0.476		
LD7-08	Dump	Lab		0.073		0.195	0.195	0.043								0.438		
LD8-08	Dump	Lab		0.077		0.149	0.154									0.314		
LG1-06	Garden	Lab	0.051	0.395		0.339	0.200				0.274			0.079	0.604	0.553	0.125	0.251
LG2-06	Garden	Lab	0.032	0.517		0.190	0.359				0.306				0.527	0.443		
LG3-06	Garden	Lab	0.346	0.455			1.802				0.528				0.928	0.337		
LG9-06	Garden	Lab		1.399							0.390				0.955	0.390		
LG4-06	Garden	Lab		0.370		0.290	0.191				0.154				0.868	0.425		
LG5-06	Garden	Lab	0.143	0.417		0.253	1.018				0.205				1.011	0.601		0.574
LG6-06	Garden	Lab	0.250	1.383		0.179	1.728								0.620	0.298		
LG7-06	Garden	Lab	0.217	0.407		0.108	2.723				0.353				0.859	0.317		0.344
LG8-06	Garden	Lab		0.694		0.200	0.200				0.216				0.283	0.355		
LD1-06	Dump	Lab		0.808												0.568		
LD2-06	Dump	Lab		1.671														
LD3-06	Dump	Lab		0.594		1.091	0.562									1.587		0.292
LD9-06	Dump	Lab		0.627												0.717		
LD4-06	Dump	Lab		0.724												0.575		

Sample Code	*i*12:0	*i*12:0 3OH	12:1 3OH	12:1 w3c	12:1 w5c	12:1 w8c	12:1 w9c	13:0	13:0 2OH	*a*13:0	*i*13:0	*i*13:0 3OH	13:1 w3c	13:1 w6c	13:1 w7c	13:1 w8c	13:1 w9c	13-eicosynoic	14:0
LG1-08																			1.779
LG2-08		0.017						0.007		0.044	0.051		0.017	0.024					0.843
LG3-08																			2.117
LG4-08																			1.148
LG5-08																			1.369
LG6-08			0.020					0.054		0.049	0.029		0.034						1.277
LG7-08		0.028			0.020			0.050		0.046	0.044		0.037						1.541
LG8-08																			1.015
LD1-08																			1.757
LD2-08																			2.278
LD3-08																			1.617
LD4-08																			1.622
LD5-08																			1.745
LD6-08																			1.344
LD7-08																			1.478
LD8-08																			1.276
LG1-06																			0.748
LG2-06																			0.591
LG3-06											0.246								0.382
LG9-06																			0.498
LG4-06																			0.542
LG5-06																			1.093
LG6-06																			0.477
LG7-06																			0.597
LG8-06											0.056								0.505
LD1-06																			1.529
LD2-06																			1.456
LD3-06																			1.468
LD9-06																			1.949
LD4-06																			1.044

Sample Code	14:0 Aldehyde	*i*14:0	*i*14:0 3OH	14:1 Cis 7 DMA	*i*14:1	14:1 w3c	14:1 w5c	14:1 w6c	14:1 w7c	14:1 w8c	14:1 w9c	14:2 w6c/*a*14:0	15:0	15:0 3OH	a15:0	*i*15:0	*i*15:0 3OH	*i*15:1	15:1 w11c
LG1-08													0.281						
LG2-08		0.027			0.024				0.007		0.024	0.030	0.219						
LG3-08													0.248						
LG4-08													0.486						
LG5-08													0.279						
LG6-08		0.009			0.014						0.003		0.369						
LG7-08		0.002							0.006		0.030		0.244		0.063				
LG8-08													0.308						
LD1-08		1.393											0.901		5.890	5.117	0.699	0.227	
LD2-08		0.619											1.078		2.385	3.043	0.428	0.268	
LD3-08		0.958											1.382		4.529	4.151	0.378	0.562	
LD4-08		1.011											1.290		3.072	4.442	0.452	0.452	
LD5-08		1.458											1.094		4.360	4.398		0.326	
LD6-08		0.710											1.234		3.171	4.005	0.779	0.379	
LD7-08		0.785											0.912		4.581	4.173	0.712	0.164	
LD8-08		1.634											0.847		6.042	5.722	0.787	0.215	
LG1-06													0.228						
LG2-06													0.295						
LG3-06																			
LG9-06													0.444						
LG4-06	0.216												0.431						
LG5-06													0.321						
LG6-06																			
LG7-06													0.280						
LG8-06													0.344						
LD1-06		1.245											0.874		2.446	3.494	0.721		
LD2-06		1.240											1.240		3.935	4.744			
LD3-06		0.810											0.389		4.406	3.488			
LD9-06		2.173											0.806		3.808	3.853	0.717		
LD4-06		0.490													0.852	1.236			

Sample Code	15:1 w5c	15:1 w6c	15:1 w7c	15:1 w8c	15:1 w9c	16:0	16:0 2OH	16:0 3OH	*i*16:0	16:1 2OH	16:1 Cis 7 DMA (w 9)	*i*16:1	16:1 w3c	16:1 w5c	16:1 w7c	16:1 w7c Alc	16:1 w8c	16:1 w9c	17:0
LG1-08						20.975	0.680				1.247			0.463	0.784				0.390
LG2-08						26.015	0.459				0.401			0.357	0.870	0.121		0.162	0.486
LG3-08		0.194				19.758	0.472				1.104			0.138			0.500		0.373
LG4-08						19.576	0.495				0.437			0.158	0.894			0.200	0.815
LG5-08		0.218				25.224	0.776				0.423			0.170	0.541				0.436
LG6-08		0.137				24.911	0.606				0.586			0.380	0.540				0.489
LG7-08		0.144				22.318	0.555				0.742			0.374	0.855				0.483
LG8-08						24.362	0.603				0.620			0.188	0.500				0.514
LD1-08		0.232				20.002	0.918	0.310	2.877		1.562			0.178	4.229	0.467			0.492
LD2-08						25.015	0.558		2.286		1.063	0.168		0.543	3.234	0.558			0.558
LD3-08						14.597	1.106		3.979		0.327	0.350	0.332	0.378	3.021	0.138			0.568
LD4-08						17.926	1.197		2.886		0.532			0.492	4.535				0.678
LD5-08						15.274	0.846	0.465	3.491		0.628				6.640	0.295			0.590
LD6-08						16.421	1.013		3.598					0.386	3.936				0.676
LD7-08						23.707	0.937	0.371	2.573		1.326				2.689	0.572			0.499
LD8-08						15.368	0.836	0.440	4.732		0.363			0.297	4.655	0.198			0.704
LG1-06						14.888	0.622				0.618			0.302	0.386				0.497
LG2-06						18.129	0.791				0.401			0.570	0.538				0.548
LG3-06						18.106	0.573				0.382								0.364
LG9-06						22.699	0.928				0.350				0.538				0.484
LG4-06				0.234		15.880	0.727				0.474			0.382	0.456				0.739
LG5-06						22.171	0.765				0.376			0.232	0.382				0.403
LG6-06						17.237	0.739				0.536			0.429					0.572
LG7-06						18.987	0.896				0.624			0.290	0.371				0.470
LG8-06						17.472	0.694				0.400			0.322	0.361			0.133	0.633
LD1-06						14.261	0.939	0.677	1.944		1.158				3.276	0.393			
LD2-06						21.456			3.450						4.582				
LD3-06						10.334	0.993	0.270	1.587		0.713		0.400	0.324		0.162			0.248
LD9-06						16.241	1.008	0.762	2.800		0.694				4.212				0.470
LD4-06						13.254			0.724						2.536				

Sample Code	*a*17:0	*cy*17:0	*i*17:0	*i*17:0 3OH	17:1 w6c	17:1 w8c	18:0	18:0 10 Me	18:0 2OH	18:1 (w ?) Alc	18:1 2OH	18:1 w5c	18:1 w7c	18:1 w9c	18:1 w9t Alc (T:17:0	18:3 w6c	19:0	19:0 10Me	19:0 2OH
LG1-08	0.156					0.476	1.623		0.558			25.469		13.581			0.294	0.351	
LG2-08	0.121					0.526	4.063		0.536		0.280				0.742		0.165	0.145	
LG3-08	0.221					0.491	1.372					22.857				0.092	0.176	0.286	0.111
LG4-08						0.931	4.262		0.453								0.166	0.133	
LG5-08	0.122					0.523	1.927		0.977		0.235					0.113	0.148	0.157	
LG6-08	0.174					0.700	1.375		0.563		0.277	6.799					0.226	0.189	
LG7-08	0.185					0.540	2.498				0.326	14.904				0.078	0.168	0.266	
LG8-08	0.137					0.692	1.553		1.004			8.895					0.199	0.590	
LD1-08	1.546	2.526	0.463	1.236		0.595	2.579	0.471	0.748		0.368			14.083		0.434	0.219	0.405	
LD2-08	0.956	1.376	1.047	0.375		0.528	2.691	0.428	0.581	0.742				19.664		0.405	0.184		
LD3-08	1.216	2.557	0.883	0.837		1.015	1.565	0.504	0.820	0.780				11.547	0.854	1.003	0.149		
LD4-08	0.864	2.912	1.024	0.692		0.758	3.125	0.731	0.519	0.931			3.058	13.218	1.223		0.359		
LD5-08	1.109	1.939	0.745	0.946		0.892	2.242	0.318	0.853				4.026	11.046	1.590	0.310	0.287		
LD6-08	1.075	2.461	0.965	0.917		0.772	2.378	0.807	0.724	0.731			2.309	13.960	0.586	0.724	0.207		
LD7-08	1.527	2.281	0.322	1.989		0.554	2.482	0.462	1.065					15.671	3.084	0.359	0.201	0.316	
LD8-08	1.469	2.284	1.018	1.865		0.704	1.942	0.374	0.792				6.146	6.988	1.056	0.352	0.171		
LG1-06	0.242					0.214	2.801		0.451		0.242	29.292		13.712		0.186	0.269	0.060	
LG2-06						0.285	2.869				0.865			9.059	0.422		0.506		
LG3-06							1.738								0.309		0.328	0.136	
LG9-06						0.336	2.839		1.023					23.991	0.458		0.888		
LG4-06	0.160	0.339	0.197			0.493	3.437		0.536		0.228			18.726			0.228		
LG5-06						0.266	2.343		0.649		0.280			14.924	0.396		0.321		
LG6-06						0.358	2.754		0.942					13.589			0.918		
LG7-06	0.271					0.262	2.786		0.823		0.208						0.235		
LG8-06	0.155		0.211			0.522	3.025		0.566		0.272					0.117	0.505		
LD1-06	0.764	1.922	0.524	1.158		0.721	1.398		1.026				2.533	3.996	1.987		0.480		
LD2-06		2.911		1.617			2.048						3.720	11.159	2.318		1.078		
LD3-06	0.885	2.268		1.447			0.767		0.821				2.030		1.058		0.205		
LD9-06	1.030	2.038	0.538	1.501		0.515	1.254		0.829						1.344		0.403		
LD4-06		0.597		0.724			5.029		0.511					39.058		0.703	0.490		

Sample Code	*cy*19:0	*cy*19:0 11-12 2OH	*i*19:0	19:0 N Alc	19:1 w11c	19:1 w5c	19:1 w8c	20:0	*i*20:0	20:1 w5c	20:1 w6c	20:1 w9c	20:2 w6c	20:4 w6c	20:5 w3c	24-Dimethyl 14:0	2-Me-12:0	2-Me-14:0	a17:1 AT9
LG1-08											0.541								
LG2-08								0.138									0.007		
LG3-08								0.089			0.192								
LG4-08								0.179											
LG5-08								0.188											
LG6-08								0.054			0.129								
LG7-08			0.061					0.111			0.311								
LG8-08											0.188								
LD1-08	1.170										0.356								0.149
LD2-08	1.529						0.260	0.145					0.290	3.211					
LD3-08	1.622	0.160	0.212				0.126	0.206						1.749					
LD4-08	2.460							0.293						1.489					
LD5-08	1.078							0.380						1.396					
LD6-08	1.972	0.083						0.152					0.076	1.441					
LD7-08	1.229																		
LD8-08	1.810		0.160					0.127						1.838					
LG1-06								0.348			0.441								
LG2-06											2.521								
LG3-06											1.192								
LG9-06																			
LG4-06								0.955			0.296								
LG5-06											0.854								
LG6-06											1.156								
LG7-06																			
LG8-06											0.638								
LD1-06	1.332													1.507					
LD2-06	1.779													1.563					
LD3-06	1.609										0.864								
LD9-06	1.210													1.142					
LD4-06								0.490						1.449					

Sample Code	C10 Primary Alc	C12 Primary Alc	C14 N Alc	C20 N Alc	C9 Dicarboxy acid	cis 910 epoxy 18:0	i17:1AT9 (T:i17:1w 9c)	i17:1 G (T:16:0 10Me)	Sebacic C10 dicarbox	Suberic C8 dicarbox	SiF 1	SiF 2	SiF 3	SiF 4	SiF 7	SiF 9	SiF 11	SiF 12	SiF 13
LG1-08		0.229		0.433	0.935					0.147						0.225	0.372		
LG2-08		0.438		0.610	0.512	0.118			0.030	0.088	0.014				27.249	0.512	0.155		
LG3-08				0.686	0.659					0.162					23.551	0.470	0.259		
LG4-08		0.437		0.503	0.653					0.166	0.204				27.771		0.183		
LG5-08				0.863	0.654	0.118				0.174					34.942	0.846	0.214		
LG6-08		0.031		0.692	0.640				0.057	0.123		0.066			25.529	0.592	0.112		
LG7-08		0.228		0.474	0.709				0.050	0.148		0.080			21.604	0.296	0.207		
LG8-08				1.025	0.514					0.079					18.832	0.473	0.315		
LD1-08				0.227	0.496							0.475	2.856				0.294		
LD2-08					0.336												0.206		
LD3-08				0.545	0.946	0.304	2.047			0.292		0.218	1.324	0.241			0.384		
LD4-08				0.505	0.918		2.673										0.359		
LD5-08	0.031			0.566	0.590		0.970										0.334		
LD6-08				0.352	0.641		1.765			0.193		0.234		0.317			0.310		
LD7-08					0.420							0.426	3.425				0.359		
LD8-08					0.297		2.449					0.396		0.369			0.209		
LG1-06	0.056	0.669		0.711	1.273	0.539			0.121	0.172	0.028					0.288			
LG2-06		0.907		3.681	0.918					0.211					8.521	0.485			
LG3-06				0.464	0.537	0.528									30.616				
LG9-06		0.646		0.444	0.619											0.458			
LG4-06		1.029		0.918	0.795	0.154				0.123	0.419					0.290			
LG5-06				0.499	0.690	0.717										0.321			
LG6-06				0.954	1.156	0.477				0.203									
LG7-06		0.163		0.534	0.516	0.298									21.158	0.507			
LG8-06	0.033	1.299		0.810	0.716	0.350				0.128	0.733				19.859	0.400			
LD1-06					0.590			1.572					1.878		2.861				
LD2-06						1.132							3.288						
LD3-06				0.767	0.983	0.745				0.292			6.803		3.304		0.410		
LD9-06							0.717	0.717				0.784	2.867		12.366				
LD4-06				2.046		2.110							1.662						

Sample Code	SiF 14	SiF 16	SiF 17	SiF 18	SiF 19	SiF 21	SiF 22	SiF 23	T:11 Me 18:1 w7c	T:Unk 16.582	Unk 18.473 (T:19:1 iI)	Unk 11.097	Unk 11.541 B	Unk 12.112	Unk 12.486	Unk 12.553 B	Unk 13.566 B	Unk 14.503	Unk 15.176
LG1-08					25.209	0.390	0.502				0.255								
LG2-08			0.084		31.049	0.236					0.378							0.115	
LG3-08	0.078		0.108		19.946	0.219					0.292							0.057	
LG4-08					37.231	0.183					0.233		0.769	0.245					
LG5-08			0.113		24.561	0.144					0.436								
LG6-08			0.094		29.190	0.180					0.260				0.014			0.077	
LG7-08	0.137		0.072		26.556	0.281					0.307				0.035			0.078	
LG8-08	0.158				34.837	0.240					0.322							0.093	
LD1-08	0.157		0.190		18.266	0.492	0.409		0.157		0.178								
LD2-08					19.342	0.352	0.635				0.306								
LD3-08					24.280	0.252	0.648		0.258		0.837								
LD4-08					18.856		0.572		0.465		0.692								
LD5-08					23.117	0.380	0.636		0.310		0.869								
LD6-08					24.031		0.503		0.379		0.579								
LD7-08	0.274				16.024	0.566	0.310												
LD8-08					20.628	0.193	0.501		0.512		0.539								
LG1-06	0.116				15.338	0.172			0.441		0.539				0.032				
LG2-06					23.128				0.675		0.559								
LG3-06					10.700	0.218			0.300		0.619								
LG9-06					24.220				0.296				1.090						
LG4-06					24.418				0.216		0.474		1.756						
LG5-06					19.282				8.176		0.519								
LG6-06					21.338				0.393		0.513								
LG7-06					25.382				0.308		0.615								
LG8-06					24.144	0.205			0.166		0.583		2.364	0.366					
LD1-06					9.150		0.437		0.699		0.961								
LD2-06					15.256				1.240										
LD3-06					5.172				1.188										
LD9-06					16.263				0.650		0.560								
LD4-06					8.971				3.282		1.449								

Sample Code	Unk 15.273 D	Unk 17.300	Unk 18.197	Unk 19.521	Unk 20.241 C	Unk 20.343 D	Unk 9.521 B
LG1-08							
LG2-08				0.094			
LG3-08					0.189		0.019
LG4-08							
LG5-08							0.017
LG6-08							0.020
LG7-08							
LG8-08							
LD1-08							0.012
LD2-08							
LD3-08							0.034
LD4-08							
LD5-08							
LD6-08							
LD7-08					0.231		
LD8-08							
LG1-06							
LG2-06							
LG3-06							
LG9-06							
LG4-06							
LG5-06							
LG6-06							
LG7-06							
LG8-06				0.150			
LD1-06							
LD2-06							
LD3-06							
LD9-06							
LD4-06							

Sample Code	System Component	Source	8:0 3OH	9:0	9:0 3OH	10:0	10:0 3OH	*i*10:0	11:0	11:0 2OH	11:0 3OH	11:0 DiMethyl Acetal	*i*11:0	*i*11:0 3OH	11:1 2OH	12:0	12:0 2OH	12:0 3OH
LD5-06	Dump	Lab		0.880														
LD6-06	Dump	Lab		0.571		0.415	0.363									0.766		
LD7-06	Dump	Lab		0.892		0.710	0.882	0.136			0.318		0.100	0.255		0.828		0.264
LD8-06	Dump	Lab		0.491		0.491										0.673		
FG9a	Garden	Field	0.126	0.124		0.050	0.578				0.041		0.059	0.034		0.501		0.038
FG9b	Garden	Field		0.081			0.168									0.286		
FG11a	Garden	Field		0.032			0.584						0.168			0.373		
FG11b	Garden	Field		0.047		0.073	1.111			0.033	0.026		0.127	0.078		0.440		0.094
FG12a	Garden	Field	0.285	0.084		0.100	1.787						0.060			0.463		0.097
FG12b	Garden	Field	0.129	0.084		0.108	0.520						0.042	0.033		0.460		0.051
FG13a	Garden	Field	0.233	0.158	0.047	0.161	1.119				0.025		0.215	0.043		0.570		0.165
FG14a	Garden	Field	0.177	0.158		0.098	1.188						0.034			0.572		0.075
FG14b	Garden	Field	0.211	0.252		0.149	0.806				0.058		0.095	0.037		0.637		0.074
FD9a	Dump	Field		0.090		0.116							0.083			0.333		
FD9b	Dump	Field		0.067		0.101	0.073		0.067	0.118	0.050					2.026		0.050
FD9c	Dump	Field		0.104		0.215	0.187									0.514		
FD9d	Dump	Field		0.139		0.312	0.289									0.937		0.254
FD11a	Dump	Field		0.074	0.074	0.222	0.183			0.070	0.046		0.346	0.219		0.423		0.138
FD11b	Dump	Field		0.136									0.209			0.462		
FD11c	Dump	Field		0.190									0.602			0.464		
FD11d	Dump	Field		0.136		0.222							0.188			0.657		
FD11e	Dump	Field		0.078												0.761		
FD12a	Dump	Field		0.092		0.198	0.150						0.136	0.136		0.547	0.087	0.194
FD12b	Dump	Field		0.196		0.190				0.550		0.183	0.170			0.321		
FD12c	Dump	Field		0.200		0.138				0.785			0.365			0.310		
FD12d	Dump	Field		0.312		0.136				0.224		0.156	0.555			0.496	0.127	
FD13a	Dump	Field		0.139		0.229	0.278				0.049		0.486	0.339		0.650	0.074	0.208
FD13b	Dump	Field		0.127		0.119	0.258				0.106		0.221	0.176		0.352		0.131
FD13c	Dump	Field		0.159		0.096	0.239				0.422		0.202	2.022		0.485		
FD13d	Dump	Field		0.210		0.128					0.228		0.173		0.524	0.584		1.231
FD13e	Dump	Field		0.084		0.168	0.242				0.049		0.301	0.212		0.528		0.212
FD14a	Dump	Field	0.043	0.136		0.122	0.157				0.064		0.111	0.147		0.562	0.082	0.129

Sample Code	*i*12:0	*i*12:0 3OH	12:1 3OH	12:1 w3c	12:1 w5c	12:1 w8c	12:1 w9c	13:0	13:0 2OH	*a*13:0	*i*13:0	*i*13:0 3OH	13:1 w3c	13:1 w6c	13:1 w7c	13:1 w8c	13:1 w9c	13-eicosynoic	14:0
LD5-06																			2.138
LD6-06																			1.635
LD7-06						0.164													0.955
LD8-06																			1.360
FG9a			0.007					0.045		0.115	0.070								0.328
FG9b										0.031									0.216
FG11a																			0.521
FG11b			0.033		0.044	0.047		0.061		0.044	0.010					0.014			0.750
FG12a										0.072									0.601
FG12b								0.051		0.069	0.030								0.631
FG13a			0.029					0.086		0.115	0.036								0.749
FG14a										0.051									0.938
FG14b								0.070		0.091	0.074								0.658
FD9a			0.106			0.100					0.113								1.041
FD9b	0.191		0.056					0.368	0.093	0.222	0.606						0.101		1.936
FD9c				0.153				0.222			0.153								2.006
FD9d			0.243																2.626
FD11a	0.049	0.007	0.088		0.049	0.081		0.159	0.106	0.070	0.236	0.183			0.035				1.548
FD11b			0.354		0.272	0.299													2.058
FD11c																			1.214
FD11d																			2.108
FD11e																			1.922
FD12a								0.140	0.053	0.121	0.203	0.048							1.176
FD12b					0.871		0.445				0.190								1.651
FD12c					1.061		0.400												1.164
FD12d			0.195		0.224		0.127	0.253		0.097	0.068							0.720	0.935
FD13a	0.168		0.078			0.118		0.237	0.070	0.184	0.258	0.053		0.041					1.455
FD13b	0.066		0.398		0.152	0.529		0.090	0.041	0.053	0.135	0.033							1.299
FD13c	0.485	0.365	1.208		2.165	1.511		0.176	0.256	0.199	0.352								1.696
FD13d	0.232	0.410	0.698		0.634			0.346	0.670	0.296	0.333	0.720			0.137				1.464
FD13e	0.084					0.109		0.198		0.133	0.282								1.550
FD14a	0.075	0.022	0.064			0.061		0.104	0.025	0.079	0.200	0.022							1.020

Sample Code	14:0 Aldehyde	*i*14:0	*i*14:0 3OH	14:1 Cis 7 DMA	*i*14:1	14:1 w3c	14:1 w5c	14:1 w6c	14:1 w7c	14:1 w8c	14:1 w9c	14:2 w6c/*a*14:0	15:0	15:0 3OH	a15:0	*i*15:0	*i*15:0 3OH	*i*15:1	15:1 w11c
LD5-06		3.521											1.006		5.093	5.564	1.415		
LD6-06		1.246											0.545		2.712	2.803	0.493		
LD7-06		0.619											0.291		3.293	2.192			
LD8-06		1.374											0.673		5.873	4.387	0.673		
FG9a												0.090	0.228						
FG9b													0.614						
FG11a													0.931						
FG11b		0.026				0.017		0.047			0.056	0.054	0.548						
FG12a													0.426						
FG12b		0.003											0.517						
FG13a		0.022									0.050		0.839		0.029	0.075			0.047
FG14a											0.079		0.405						
FG14b											0.029		1.017		0.074	0.203			
FD9a		0.512	0.086										0.592		1.114	2.322		0.389	
FD9b		0.525	0.059		0.017		0.356		2.551		0.634	0.623	1.308		0.679	1.889		0.261	
FD9c		0.736											1.180		1.464	5.608		0.298	
FD9d		0.706											1.145		1.249	2.822			
FD11a		2.024	0.064	0.056			0.138		0.402		0.070	0.201	1.587		3.033	4.658	0.243	0.561	
FD11b		0.671											1.451		1.270	4.126			
FD11c		0.560											1.108		1.151	2.850			
FD11d		0.503											1.007		1.945	3.532			
FD11e		0.588											0.847		1.585	4.158		0.165	
FD12a		1.108							0.106		0.048		1.016		2.783	3.683	0.261	0.271	
FD12b		0.707									0.386		0.910	0.360	1.624	4.638			
FD12c		0.661									0.524		0.827		1.667	3.672			
FD12d	0.058	0.448									0.117	0.097	0.964		0.720	0.652			
FD13a		1.095	0.139	0.029			0.098		0.630		0.106	0.196	1.467		4.284	3.920	0.196	0.396	
FD13b		0.639	0.602				0.016				0.070		1.930		1.508	3.212		0.119	
FD13c		1.544	0.252	0.385						1.036			1.650	0.136	1.013	1.932		0.418	
FD13d		1.532		0.534									2.403		1.865	3.493			
FD13e		1.254							0.494			0.153	1.358		4.187	4.646	0.202	0.607	
FD14a		0.748			0.054		0.050		0.150		0.068	0.125	1.331		2.132	2.912	0.136	0.161	

Sample Code	15:1 w5c	15:1 w6c	15:1 w7c	15:1 w8c	15:1 w9c	16:0	16:0 2OH	16:0 3OH	*i*16:0	16:1 2OH	16:1 Cis 7 DMA (w 9)	*i*16:1	16:1 w3c	16:1 w5c	16:1 w7c	16:1 w7c Alc	16:1 w8c	16:1 w9c	17:0
LD5-06						13.581	1.509	1.132	4.087						4.370				
LD6-06						14.612	0.792		1.960		0.584				3.322				0.519
LD7-06						9.270	0.682		1.337		0.655				2.566				0.246
LD8-06						12.363	1.135	0.617	4.402		0.743				3.336	0.322			0.519
FG9a						25.207	0.178				0.192			0.172	0.115			0.154	0.556
FG9b						31.583	0.415				0.182			0.406	0.552			0.109	0.451
FG11a						24.646	0.442				0.331			0.657	0.342	0.132		0.216	1.178
FG11b						17.191	0.303				0.312			0.352	0.334	0.082		0.313	1.161
FG12a						17.772	0.482				0.441			0.326	0.250	0.081		0.260	0.745
FG12b						21.882	0.438				0.595			0.318	0.261			0.264	0.775
FG13a		0.050	0.047			23.702	0.552				0.262			0.703	0.298	0.036		0.265	0.979
FG14a						14.551	0.271				0.194			0.252	0.322	0.034		0.390	0.953
FG14b						24.897	0.649				0.492			0.724	0.269	0.116		0.203	0.959
FD9a						12.023	0.316		0.922		0.459			1.268	3.127	0.113			0.349
FD9b		0.511		0.168		12.550	0.222		1.094		0.553	0.087		0.884	4.058	0.244			1.120
FD9c						15.387	0.874		1.798		0.951			5.566	3.290	0.326			0.916
FD9d						18.913	0.671		4.673		0.972	0.578		0.763	2.082	0.497			0.902
FD11a		0.286			0.074	15.844	0.254	0.212	2.278		0.314			0.677	9.838	0.130			1.100
FD11b						17.728	0.635		2.058		0.698			5.232	3.101	0.254			3.246
FD11c						15.634	1.003	0.306	3.294		0.633	0.517							0.866
FD11d						15.913	0.478		3.063		1.544			0.555	1.374	0.666			0.819
FD11e						16.469	0.620		2.126	0.392	0.471	0.408		3.107	2.370	0.314		0.204	0.769
FD12a		0.121				18.209	0.954	0.237	2.120		0.600			0.532	9.080	0.116			1.133
FD12b						13.677	0.681		2.011		0.891	0.321		4.611	1.801	0.524		0.334	0.825
FD12c						12.909	0.689		2.287		0.820			0.985	2.590	0.441			0.799
FD12d		0.166			0.088	22.656	1.752		3.038		0.273	0.350		0.127	0.545	0.175			0.769
FD13a	0.012	0.151		0.057	0.070	16.076	1.022	0.298	2.326		0.740			0.732	6.078	0.196			2.174
FD13b		0.438				14.653	1.233		3.004		0.647	1.512		4.716	4.306			2.176	2.680
FD13c		0.478		0.704		10.162	0.305	0.053	0.950	0.110	0.206	0.720		1.829	1.607	0.199		0.362	0.561
FD13d		1.149			0.511	9.228	0.460		2.084	0.169	0.720	0.948		3.046	2.790	0.638			0.921
FD13e		0.123				19.114	0.573	0.286	2.666		0.356			1.012	7.145	0.158		0.262	0.780
FD14a		0.107				17.380	0.580	0.172	1.449		0.386			0.855	6.891				0.973

**Sample Code**	***a*17:0**	***cy*17:0**	***i*17:0**	***i*17:0 3OH**	**17:1 w6c**	**17:1 w8c**	**18:0**	**18:0 10 Me**	**18:0 2OH**	**18:1 (w ?) Alc**	**18:1 2OH**	**18:1 w5c**	**18:1 w7c**	**18:1 w9c**	**18:1 w9t Alc (T:17:0)**	**18:3 w6c**	**19:0**	**19:0 10Me**	**19:0 2OH**
LD5-06	1.446	3.678	0.692	2.578			1.226		1.352				2.955	4.841	0.912		0.660		
LD6-06	0.921	1.233	0.467	0.714		0.389	2.466		0.597				2.972	10.719	0.934	0.311	0.324		
LD7-06	0.691	1.210	0.500	0.473		0.409	0.901		0.509				2.020		1.046	0.173	0.209		
LD8-06	1.780	1.808	0.491	1.892		0.491	1.206		1.009				4.612	2.481	1.402		0.350		
FG9a	0.099					0.305	24.902				0.610				0.108		0.156		
FG9b						0.148	18.144				0.552	1.398					0.227		
FG11a						0.342	6.606		1.283		0.268	4.344		18.671	0.810		0.258		
FG11b	0.090	0.275				0.488	7.813				0.232	1.894			0.397	0.101	0.218		
FG12a	0.078					0.285	7.468		0.955		0.379				0.385		0.150		
FG12b	0.030	0.102				0.279	6.520				0.393				0.556		0.147		
FG13a	0.043	0.136				0.272	6.652				0.681	3.249			0.420		0.251		
FG14a	0.045					0.442	9.668				1.071	0.915				0.171	0.188		
FG14b	0.141					0.372	7.009				0.773			16.362			0.285		
FD9a	0.359	0.689	0.412	0.160		0.316	3.084	0.103	0.333		0.206	5.672				0.216	0.136		
FD9b	0.466	0.617	0.657			0.814	3.732	0.244	0.236			6.157			1.221	0.135	0.135		
FD9c	0.638	1.416	1.576	0.382		0.458	3.109	0.153	0.847	0.285		7.961	2.707	4.421	1.610		0.180		
FD9d	1.191		1.041		1.458	0.289	4.176	1.157	1.053			10.931	1.353	5.599	2.533		0.266		
FD11a	0.793	1.354	0.631	0.458		1.044	2.641	0.152	0.451		0.201		3.569	5.381	0.547	0.367	0.134		
FD11b	1.288	2.711	1.714	0.236		0.898	3.065	0.707	1.061				2.448	5.958	1.333	0.218	0.190		
FD11c	1.700	1.510	0.802	0.992		0.454	4.180	1.003	1.900				1.541	5.479	1.024	0.158	0.264		
FD11d	1.416	1.886	1.655			0.495	3.430	1.297	0.589	0.427			1.971	4.096	3.089		0.222		
FD11e	1.036	1.522	1.506			0.424	3.978	0.612	0.400						1.452	0.581	0.149		
FD12a	0.949	1.370	0.368	0.842		0.837	2.430	0.184	1.205		0.344		1.796	9.250	0.532	0.155	0.155		
FD12b	1.002	1.611	2.240	0.177		0.308	2.358	0.504	0.452	0.675			1.624	4.454	2.561		0.124		
FD12c	1.274	1.612	1.481	0.124		0.413	2.494	0.799	0.462	0.668			1.488	5.786	2.039		0.152		
FD12d	1.032	0.555					3.369	1.022	2.385					5.326	1.013		1.217		
FD13a	1.357	1.917	0.552	0.527		0.805	2.841	0.164	0.764		0.253		3.617	4.807	0.875		0.110		0.196
FD13b	1.086	2.602	1.549	0.299		0.951	4.180	0.135	0.754		0.209	2.897	3.581	5.847	0.652	0.369	0.188		
FD13c	0.558	0.820	0.618	0.080		0.349	2.507	0.120	0.329	0.083	0.073		1.321	7.161	0.252	0.422	0.086		
FD13d	1.076		1.559	0.132	1.322	0.337	1.883	0.506	0.556	0.027	0.342		1.532	4.746	0.761	0.214	0.438		
FD13e	1.106	1.062	0.528	0.988		0.518	3.614	0.212	0.706		0.133		4.726	8.429	0.721		0.089		
FD14a	0.730	1.059	0.404	0.512		0.644	3.570	0.136	0.794		0.272		3.556	7.198	0.669	0.315	0.182		

Sample Code	*cy*19:0	*cy*19:0 11-12 2OH	*i*19:0	19:0 N Alc	19:1 w11c	19:1 w5c	19:1 w8c	20:0	*i*20:0	20:1 w5c	20:1 w6c	20:1 w9c	20:2 w6c	20:4 w6c	20:5 w3c	24-Dimethyl 14:0	2-Me-12:0	2-Me-14:0	a17:1 AT9
LD5-06	2.075													1.383					
LD6-06	0.856							0.363			0.363			1.739					
LD7-06	1.046										0.919			0.500					
LD8-06	0.743							3.210			0.659			0.883					
FG9a	0.124		0.140					3.528				0.102	0.280						
FG9b	0.210		0.140					2.115					0.151						
FG11a								0.815					0.158						
FG11b								0.622				0.108	0.303	0.045					
FG12a			0.194					0.635					0.473						
FG12b			0.120					0.637				0.129	0.393						
FG13a	0.136		0.258					0.620					0.262					0.090	
FG14a	0.100		0.113			0.062		0.608				0.181	0.333						
FG14b	0.442		0.281					1.121					0.050						
FD9a	1.221							0.669				0.133		0.336					
FD9b	1.134	0.138			0.124			0.368				0.390		0.581			0.048	0.034	
FD9c	2.971	0.569					0.194	0.694	0.305				0.083	0.882					
FD9d	2.834							1.515	0.405					0.370					
FD11a	0.434							0.529					0.254	1.908				0.046	
FD11b	1.968	0.209					0.163	1.179						1.514					
FD11c	5.415	0.475						3.906											0.401
FD11d	3.874	0.375						1.425						0.674					
FD11e	2.966	0.188						0.777						0.902					
FD12a	0.344		0.184					0.552					0.165	0.562					
FD12b	2.607	0.223					0.236	0.622					0.203	0.622					
FD12c	2.308			0.379				1.047						0.937					
FD12d	1.081							1.558		1.052					0.779	0.039		0.010	
FD13a	0.589		0.135					1.153						0.315					
FD13b	1.692	0.168	0.135					1.479					0.291	1.610	0.410				
FD13c	0.916	0.103						0.893				0.073		0.329					
FD13d	2.093	0.356	0.141				0.310	0.602				0.132	0.164	0.802					
FD13e	0.637							0.849					0.035	0.795					
FD14a	0.261		0.079					0.504					0.161	1.313					

Sample Code	C10 Primary Alc	C12 Primary Alc	C14 N Alc	C20 N Alc	C9 Dicarboxy acid	cis 910 epoxy 18:0	*i*17:1AT9 (T:*i*17:1w 9c)	*i*17:1 G (T:16:0 10Me)	C10 Sebacic dicarbox	C8 Suberic dicarbox	SiF 1	SiF 2	SiF 3	SiF 4	SiF 7	SiF 9	SiF 11	SiF 12	SiF 13
LD5-06						1.006	1.572						5.376						
LD6-06				0.389	0.571	1.479	0.662	0.675				0.519	1.583						
LD7-06				0.455	0.737	0.218		1.310		0.218			1.328		5.868		0.291		
LD8-06					0.757		0.785	0.883					4.275				0.420		
FG9a	0.014	0.020	0.029	0.856	0.827				0.090	0.142	0.011				17.704	0.124			0.287
FG9b				0.908	0.524					0.123					20.856				0.140
FG11a				1.262	0.410					0.110									
FG11b				1.565	0.246				0.035	0.071					25.209	0.077	0.061		0.160
FG12a				1.427	0.623				0.094	0.182					23.522	0.210			0.250
FG12b		0.024		1.192	0.468				0.063	0.135					23.258	0.201			0.264
FG13a				1.621	0.749				0.111	0.244					19.980				0.118
FG14a				1.499	0.314				0.090	0.102					29.831				0.132
FG14b				1.894	0.951				0.186	0.397									
FD9a					0.479					0.103					16.361	0.306			
FD9b				0.177	0.410				0.073	0.107					27.895	0.446			
FD9c					0.743			2.533		0.229		7.406							
FD9d					1.851			2.695	0.289	0.486		0.532							
FD11a		0.021		0.444	0.522		1.421		0.018	0.180		0.190		0.233					
FD11b				0.399	1.152			2.965	0.227	0.326							0.689		
FD11c					2.840			1.214	0.158	0.665			3.125					1.161	
FD11d				0.324	1.280			3.618	0.145	0.307			0.572				1.186		
FD11e				0.345	0.518			3.429							22.111	0.369			
FD12a				0.402	0.823		0.760	0.639		0.179		0.305					0.194		
FD12b			0.242	0.223	0.596			3.157											
FD12c				0.386	0.751			2.094	0.227										
FD12d				0.604	2.230			0.292	0.166	0.672		0.146	0.496						
FD13a				0.405	1.038		0.956		0.057	0.172		0.511		0.711					
FD13b					0.955				0.090	0.156									
FD13c					1.852	0.120				0.262						0.189	0.491		0.040
FD13d				0.141	1.414			2.389	0.743	0.274				0.388			0.871		
FD13e				0.296	0.617		0.706	1.032		0.138		0.464					0.193		
FD14a		0.036		1.027	0.683		0.572		0.125	0.125		0.204					0.161		0.186

Sample Code	SiF 14	SiF 16	SiF 17	SiF 18	SiF 19	SiF 21	SiF 22	SiF 23	T:11 Me 18:1 w7c	T:Unk 16.582	Unk 18.473 (T:19:1il)	Unk 11.097	Unk 11.541 B	Unk 12.112	Unk 12.486	Unk 12.553 B	Unk 13.566 B	Unk 14.503	Unk 15.176
LD5-06					15.121				1.069										
LD6-06					9.525		0.649		1.972		2.037								
LD7-06					7.214	0.264			0.519		0.418								
LD8-06					11.831	0.378	0.505		0.350		0.631								
FG9a		0.002	0.054		13.945						0.291				0.034		0.014	0.016	
FG9b					14.894														
FG11a					27.628						0.300								
FG11b			0.162		29.279	0.108					0.425				0.021				
FG12a			0.122		24.984						0.714								
FG12b			0.114		24.027						0.412								
FG13a			0.090		21.178						0.244							0.054	
FG14a			0.175		28.722	0.062					0.629								
FG14b					21.026	0.157					0.198								
FD9a					37.841				0.210										
FD9b			0.286		11.551						0.084				0.020				
FD9c					7.399		0.285		0.437										
FD9d					9.774													0.208	
FD11a			0.215		12.959	0.219	0.451	0.159	0.106		0.254						0.085		
FD11b					5.495		1.016		0.326										
FD11c					7.400	3.241	2.322		0.654										
FD11d					5.136	0.410	0.316		0.401										
FD11e					8.552		0.377		0.541										
FD12a			0.198		14.380						0.121								
FD12b				0.341	3.006				0.367										
FD12c					4.367		0.227		0.227										
FD12d		0.487	0.068		5.326	0.964	2.327							0.419			0.185		
FD13a			0.282		8.804	0.131		0.098	0.110						0.037		0.106		
FD13b					9.658		0.578	0.291	0.381						0.106		0.066		
FD13c			0.266	0.070	10.806	0.080	0.458	0.375	0.226	0.130	0.007	0.090			0.581	0.428			0.594
FD13d	0.023		0.524	0.091	4.382	0.378	0.374	0.506	0.365	0.169		0.068			0.292	0.292	0.611		
FD13e			0.262		11.737	0.153	0.346		0.193		0.123								
FD14a			0.150		21.870	0.157	1.184		0.100		0.318				0.025				

Sample Code	Unk 15.273 D	Unk 17.300	Unk 18.197	Unk 19.521	Unk 20.241 C	Unk 20.343 D	Unk 9.521 B
LD5-06							
LD6-06							
LD7-06							
LD8-06							
FG9a				0.160			0.007
FG9b							
FG11a				0.216			
FG11b				0.327		0.162	
FG12a				0.391			
FG12b				0.466			0.066
FG13a				0.194			0.054
FG14a				0.461			
FG14b							0.116
FD9a						0.313	0.096
FD9b	0.017					0.269	0.048
FD9c						0.618	0.146
FD9d						0.706	
FD11a	0.011						0.106
FD11b							
FD11c							
FD11d							0.179
FD11e							
FD12a							0.077
FD12b		0.183					0.164
FD12c						0.406	
FD12d	0.049						0.146
FD13a	0.024		0.507			0.507	0.082
FD13b						0.258	0.082
FD13c	0.242					0.183	0.070
FD13d	0.483						0.087
FD13e							
FD14a							0.100
